# Disparity of smell tests in Alzheimer’s disease and other neurodegenerative disorders: a systematic review and meta-analysis

**DOI:** 10.3389/fnagi.2023.1249512

**Published:** 2023-09-07

**Authors:** Silin Liu, Zhihui Jiang, Jing Zhao, Zhensheng Li, Ruixin Li, Yunyi Qiu, Hua Peng

**Affiliations:** ^1^Department of Otolaryngology Head and Neck Surgery, General Hospital of Southern Theater Command, Guangzhou, China; ^2^The First School of Clinical Medicine, Southern Medical University, Guangzhou, China; ^3^Department of Pharmacy, General Hospital of Southern Theater Command, Guangzhou, China; ^4^School of Pharmaceutical Sciences, Southern Medical University, Guangzhou, China; ^5^Graduate School, Guangzhou University of Chinese Medicine, Guangzhou, China; ^6^Department of Neurology, General Hospital of Southern Theater Command, Guangzhou, China

**Keywords:** Alzheimer’s disease, smell test, olfactory impairment, smell, systematic review

## Abstract

**Background:**

There are discrepancies of olfactory impairment between Alzheimer’s disease (AD) and other neurodegenerative disorders. Olfactory deficits may be a potential marker for early and differential diagnosis of AD. We aimed to assess olfactory functions in patients with AD and other neurodegenerative disorders, to further evaluate the smell tests using subgroup analysis, and to explore moderating factors affecting olfactory performance.

**Methods:**

Cross-sectional studies relating to olfactory assessment for both AD and other neurodegenerative disorders published before 27 July 2022 in English, were searched on PubMed, Embase and Cochrane. After literature screening and quality assessment, meta-analyses were conducted using stata14.0 software.

**Results:**

Forty-two articles involving 12 smell tests that evaluated 2,569 AD patients were included. It was revealed that smell tests could distinguish AD from mild cognitive impairment (MCI), Lewy body disease (LBD), depression, and vascular dementia (VaD), but not from diseases such as frontotemporal dementia (FTD). Our finding indicated that in discriminating AD from MCI, the University of Pennsylvania Smell Identification Test (UPSIT) was most frequently used (95%CI: −1.12 to −0.89), while the Brief Smell Identification Test (B-SIT), was the most widely used method in AD vs. LBD group. Further subgroup analyses indicated that the methods of smell test used contributed to the heterogeneity in olfactory threshold and discrimination scores in group AD vs. MCI. While the moderating variables including age, MMSE scores, education years in AD vs. LBD, were account for heterogeneity across studies.

**Conclusion:**

Our finding suggests smell tests have potential value in early differential diagnosis of AD. UPSIT and its simplified variant, B-SIT, are widely used methods in the analyses.

**Systematic Review Registration:**

https://www.crd.york.ac.uk/PROSPERO/display_record.php? RecordID = 357970 (PROSPERO, registration number CRD42022357970).

## Introduction

As a leading contributor to dementia, Alzheimer’s disease (AD) is a progressive neurodegenerative disorder, and currently affecting millions of people (Kivipelto et al., 2020). The Alzheimer’s Disease International (ADI) predicted that, with the process of social aging, the number of dementia patients would exceed 152 million by 2050 (Nichols et al., 2022). Preventing or even delaying the onset of AD for only a few years could substantially reduce its prevalence and related human and economic burdens (Brookmeyer et al., 1998). Since the disease-modifying therapy based on early recognition and intervention is considered to be most beneficial in prevention of AD (Serby et al., 1991; Peters et al., 2003), smell tests attracted extensive attention for its ability to pinpoint the period of AD and to discriminate AD from other disorders (Serby et al., 1991; Kivipelto et al., 2020).

Pathological changes of AD were observed in olfactory regions at an initial stage (including entorhinal cortex, olfactory bulb, and olfactory nucleus), while structural and functional abnormalities in olfactory regions had been reported in AD patients as Braak and Del Tredici (2015) mentioned. Furthermore, longitudinal researches suggested that olfactory impairment, particularly the rapid decline in olfactory identification, may predict decline of cognition and the progression of AD (Pacyna et al., 2022). Olfactory impairment also occurs in some other diseases, including neurodegenerative diseases such as Parkinson’s Disease (PD), Lewy Body Disease (LBD), and epilepsy, as well as some conditions as depression, HIV, and healthy elderly individuals (Marine and Boriana, 2014; Dan et al., 2021). In fact, smell tests were applied in early differential diagnosis of AD and other diseases, such as other terms of dementia including LBD, vascular dementia (VaD), and frontotemporal dementia (FTD), that can be easily intertwined with AD (Duff et al., 2002; Luzzi et al., 2007; Marine and Boriana, 2014; Westervelt et al., 2016; Yoo et al., 2018), since there may be differences in extent of olfactory impairment between AD and those conditions.

Several meta-analyses have studied olfactory impairment in AD and PD patients. Mesholam et al. conducted a meta-analysis to assess olfactory function in AD, PD, and healthy controls in three measured olfactory domains, including olfactory identification, olfactory threshold, and olfactory recognition (Mesholam et al., 1998). The results revealed severe deficits in all three olfactory domains in both patients with AD and PD when compared to health controls. However, they found there was no discriminating olfactory deficits between AD and PD groups. Another meta-analysis updated the results in the former meta-analysis and characterized AD and PD patterns of olfactory deficits (Rahayel et al., 2012). It indicated that AD and PD patients are more impaired on olfactory identification and recognition tasks than on odor detection thresholds task. Additionally, PD patients are more impaired on detection thresholds than AD patients. However, rather than directly comparing olfactory function in patients with AD and in patients with other neurological conditions, all the included studies in the above two meta-analyses evaluated olfactory function by comparing health controls with AD and PD patients, separately. In the meantime, although many researches indicated that the degree of olfactory impairment was different in AD, MCI and other neurodegenerative diseases, there was little pooled data to compare them. Therefore, it is still unknown which neurodegenerative conditions can be distinguished from AD by smell tests. Moreover, which smell tests have the ability in discrimination and whether there are any independent influencing factors, remains to be explored. Systemic quantitative studies to assess the olfactory impairment between AD and other disorders are lacking.

Consequently, we conducted a comprehensive systematic review and meta-analysis by all available cross-sectional studies on smell test scores in both AD, and other conditions. First, we included and further investigated studies that compared AD with other neurodegenerative disorders using smell tests. Then, subgroup analyses were performed based on the smell test methods used in comparing AD and other neurodegenerative conditions. Furthermore, exploratory meta-regression analyses were conducted to observe the potential influence factors on the analysis results. Some possible factors, such as age, gender, and mini-mental state examination (MMSE) score, and education years were examined to determine whether they influenced the differences between AD and other neurodegenerative disorders, particularly between AD and MCI. In order to better understand the available findings, we applied no restrictions on the methods of smell test and on the neurodegenerative disorders.

## Methods

The meta-analysis protocol was registered in the International Prospective Register of Systematic Reviews (PROSPERO, registration number CRD42022357970). Studies were selected and analyzed following the Preferred Reporting Items for Systematic Reviews and Meta-Analyses (PRISMA) recommendations (Moher et al., 2009).

### Inclusion criteria

We selected cross-sectional studies that matched the following criteria: (1) the subjects included AD patients and at least one AD matched group with other neurodegenerative disorders; (2) AD patients and their matched individuals received at least one standard psychophysical test to assess olfactory function; (3) published in English; and (4) statistical information, such as means and standard deviations (SD) on which effect sizes could be calculated, was provided.

### Exclusion criteria

Studies were excluded if: (1) they were duplicate publications, reviews, editorials, case report, or letters; (2) included subjects had comorbid neurological conditions that may also impair olfactory functions; or (3) they were deficient in valid data or available access.

### Search strategies

A comprehensive search of studies published in English was conducted up to 27 July 2022 using the following electronic databases: PubMed, Embase, and Cochrane. The search terms used were incorporated in the following expression: (smell OR olfac* OR odor*) AND (Alzheimer disease OR Alzheimer*). The search was restricted to human-related articles. This retrieval strategy necessitated a subsequent manual screening to select studies that contain both AD patients and matched groups with other neurodegenerative disorders. To identify additional studies, we manually checked the relevant references in the target literature. The specific search details for the three electronic databases were presented in Supplementary Figures 1–3.

### Data extraction and quality assessment

The data extraction quality and assessment were performed independently by two researchers (Silin Liu and Zhihui Jiang), and all disputes were settled by discussion. Relevant data were collected for our meta-analyses, including demographic characteristics (number of participants in each group, mean age, percentage of men, and mean education years), clinical diagnostic criteria, mean MMSE scores, and methods of smell tests with its scores which were denoted as mean and SD. If the results were expressed as the mean value and the standard error of mean (SEM), the SEM was converted to SD, by using the calculation formula: SD = SEM $\sqrt{n}$.

The risk of bias in the included studies was analyzed according to the revised version of the Quality Assessment of Diagnostic Accuracy Studies (QUADAS-2), to assess the quality of this meta-analysis (Whiting et al., 2011). QUADAS-2 is constituted of four parts: patient selection, index test, flow, and timing and reference standard. The risk of bias is classified as “high risk bias,” “low risk bias” and “unclear risk bias.”

### Statistical analysis

First, a descriptive analysis was carried out to summarize and further evaluate differences in relevant characteristics between individuals with AD and with other neurodegenerative conditions.

Then, meta-analyses were conducted to quantitatively assess the olfactory performance of individuals with AD and with different conditions separately. SMD, 95% confidence intervals (95% CI) and estimated Cohen’s d were calculated, respectively, in each study to describe the results. The magnitude of effect size was defined as small (0.2 ≤ d < 0.5), medium (0.5 ≤ d < 0.8), or large (d ≥ 0.8) according to the previous definition (Cohen, 1992). Subgroup analyses were also applied based on distinct methods of clinical olfactory assessment. The publication bias was assessed by Egger’s test and Duvaland Tweedie trim and fill method. We qualified the presence of heterogeneity using Cochrane’s Q-statistic and generated I^2^ to quantify the degree of heterogeneity among effect sizes (Hedges and Olkin, 1985). We assumed heterogeneity if P_Q_ was significant at *p* < 05. Sensitivity analyses were carried out with a leave-one-out model to evaluate the influence of each study on the stability of the overall pooled estimates. Furthermore, we performed meta-regression to explore potential moderators on heterogeneity in each disease group with more than five studies. All the above analyses were conducted by Stata V 14.0 statistical software.

## Results

### Study selection

**Figure 1:**
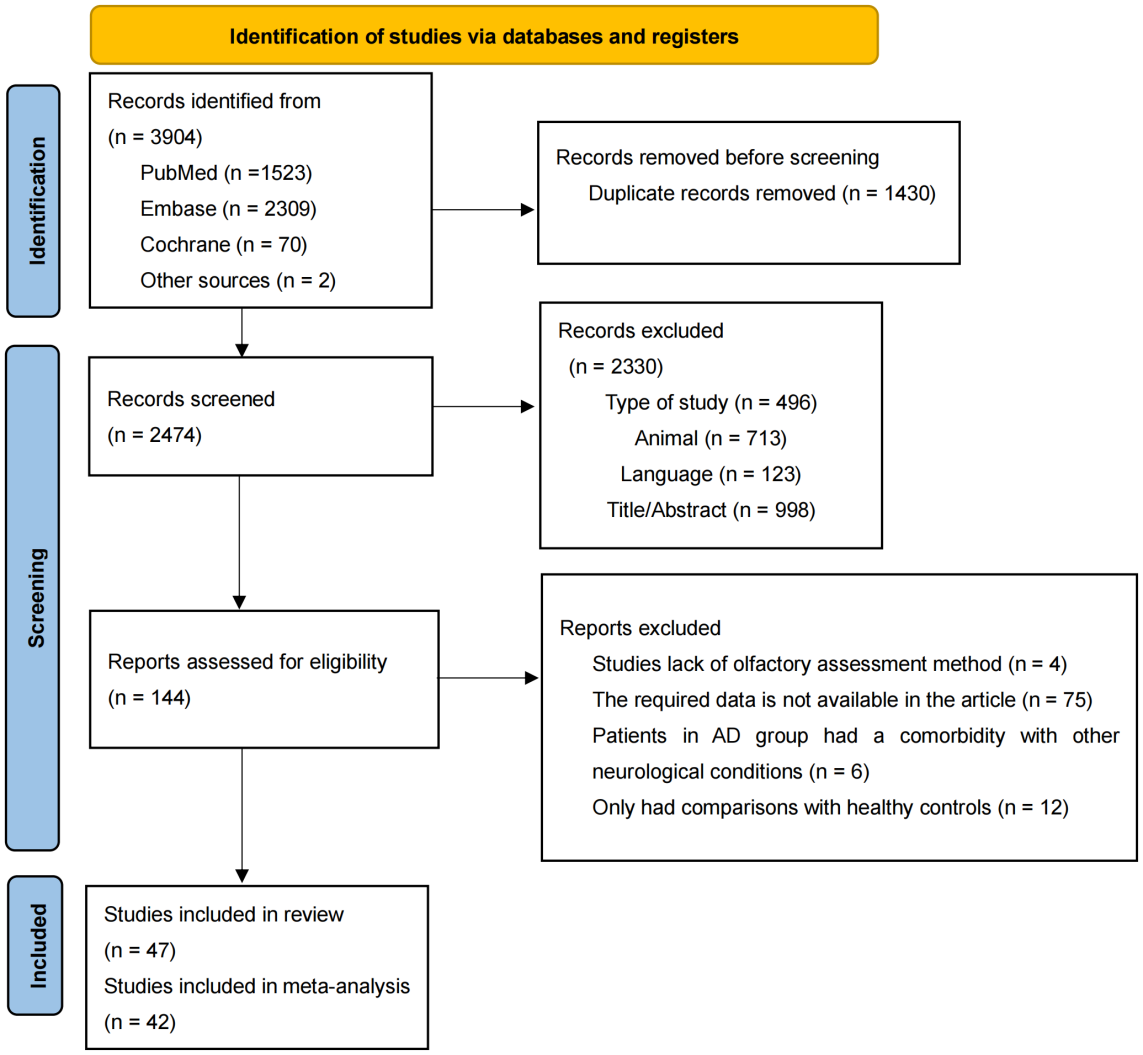
Flowchart of study selection. AD, Alzheimer’s disease.

The details of the literature selection process were presented in Figure 1. Through a preliminary literature search, 3,904 articles were included, with 1,523 articles in PubMed, 2,309 articles in Embase, 70 articles in Cochrane, and 2 articles by checking from the relevant references in the target literature. After removal of duplicate records, 2,747 articles remained. By checking titles, abstracts publication language, species, and type of study, 2,330 records were excluded for lack of relevance. Later 95 articles were excluded for the following reasons: they only had comparisons with healthy controls (*n* = 12); lack of olfactory assessment methods (*n* = 4); required data was not available (*n* = 75) and patients in AD group had comorbidity with other neurodegenerative conditions (*n* = 6). Consequently, 47 articles were considered eligible and 42 articles were included in the meta-analysis (Doty et al., 1991; Solomon et al., 1998; McCaffrey et al., 2000; Duff et al., 2002; Peters et al., 2003; Westervelt et al., 2003; Tabert et al., 2005; Motomura and Tomota, 2006; Pentzek et al., 2007; Djordjevic et al., 2008; McLaughlin and Westervelt, 2008; Westervelt et al., 2008; Williams et al., 2009; Bahar-Fuchs et al., 2010; Steinbach et al., 2010; Bahar-Fuchs et al., 2011; Sato et al., 2011; Tkalčić et al., 2011; Seligman et al., 2013; Körtvélyessy et al., 2015; Westervelt et al., 2016; Quarmley et al., 2017; Reijs et al., 2017; Umeda-Kameyama et al., 2017; Vasavada et al., 2017; Ward et al., 2017; Woodward et al., 2017; Yoo and Ye, 2017; Chen et al., 2018; Doorduijn et al., 2018; Woodward et al., 2018; Yoo et al., 2018; Wu et al., 2019; Yoshii et al., 2019; Bathini et al., 2020; Forsberg et al., 2021; Inagawa et al., 2020; Kim et al., 2020; Zhao et al., 2020; Jesmanas et al., 2021; Wang et al., 2021; Chen et al., 2022), since only one article was included in five disease types and one olfactory task, which was insufficient for the meta-analysis (Moberg et al., 1997; Luzzi et al., 2007; Naudin et al., 2014; Passler et al., 2017; Sundermann et al., 2021).

### Study characteristics

After literature selection, 44 articles involving 4,896 subjects, of which 2,569 with AD, 1,448 with MCI, 558 with LBD, 205 with depression, 44 with PD, 42 with VaD, and 30 with FTD, were qualified for this meta-analysis.

We summarized all the 12 tests in the included studies to assess olfactory functions. The function of these smell tests included three aspects: olfactory identification, olfactory threshold and olfactory discrimination. Most smell tests in this meta-analysis were for olfactory identification. These olfactory identification tests included the University of Pennsylvania Smell Identification Test (UPSIT) which consisted of 40-item version (*n* = 10; Doty et al., 1991; Tabert et al., 2005; Motomura and Tomota, 2006; Djordjevic et al., 2008; Vasavada et al., 2017; Ward et al., 2017; Woodward et al., 2017, 2018; Wu et al., 2019; Bathini et al., 2020) and 10-item version (*n* = 2; Bahar-Fuchs et al., 2010, 2011), the Sniffin’ Sticks test (SS) which consisted SS-16 version (*n* = 10; Pentzek et al., 2007; Williams et al., 2009; Steinbach et al., 2010; Seligman et al., 2013; Quarmley et al., 2017; Chen et al., 2018; Doorduijn et al., 2018; Zhao et al., 2020; Wang et al., 2021; Chen et al., 2022) and SS-12 version (*n* = 2; Körtvélyessy et al., 2015; Jesmanas et al., 2021), the Cross-Cultural Smell Identification Test (CCSIT) which was also known as the Brief Smell Identification Test (B-SIT, *n* = 8; Westervelt et al., 2003, 2008, 2016; McLaughlin and Westervelt, 2008; Reijs et al., 2017; Yoo and Ye, 2017; Yoo et al., 2018; Forsberg et al., 2021), the Odor Stick Identification Test for Japanese (OSIT-J, *n* = 3; Umeda-Kameyama et al., 2017; Yoshii et al., 2019; Inagawa et al., 2020), the Pocket Smell Test (PST, *n* = 3; Solomon et al., 1998; McCaffrey et al., 2000; Duff et al., 2002), the Open Essence Smell Identification test (*n* = 1; Sato et al., 2011), the Scandinavian Odor Identification Test (S-OIT, *n* = 1; Tkalčić et al., 2011), the YSK olfactory function test (*n* = 1; Kim et al., 2020) and the common odors (16 items; *n* = 1; Peters et al., 2003). The N-butanol alcohol test, Sniffn’ Sticks test and the YSK olfactory function test were used to assess olfactory threshold function, whereas the triplet of pens, modified version of pens, Sniffn’ Sticks test and the YSK olfactory function test were applied in olfactory discrimination examination (Peters et al., 2003; Djordjevic et al., 2008; Steinbach et al., 2010; Doorduijn et al., 2018; Kim et al., 2020). More detailed characteristics of the included studies were presented in Table 1 and Supplementary Table 1. The result of quality assessment was presented in Supplementary Table 2.

**Table 1:** Characteristics of the included studies.

Diseases	Number of smell tests:	References	Groups	OI	Index test	OT	Index test	OD	Index test
MCI	OI (*n* = 7)	Bahar-Fuchs et al. (2010)	AD (*n* = 20)	2.1 (1.1)	UPSIT-10	-	-	-	-
	OT (*n* = 4)		MCI (*n* = 24)	2.7 (1.1)		-		-	
	OD (*n* = 5)	Bahar-Fuchs et al. (2011)	AD (*n* = 25)	2.2 (1)	UPSIT-10	-	-	-	-
			MCI (*n* = 25)	2.6 (1.1)		-		-	
		Bathini et al. (2020)	AD (*n* = 17)	12 (8)	UPSIT-40	-	-	-	-
			MCI (*n* = 21)	20 (8)		-		-	
		Chen et al. (2022)	AD (*n* = 31)	6.6 (2.7)	SS-16	-	-	-	-
			MCI (*n* = 118)	10.6 (2.3)		-		-	
		Djordjevic et al. (2008)	AD (*n* = 27)	19.89 (6.5)	UPSIT-40	-	-	5.6 (3.8)	Modified version of pens
			MCI (*n* = 51)	27.25 (6.9)		-		7.9 (3.2)	
		Doorduijn et al. (2018)	AD (*n* = 30)	9.1 (0.5)	SS-16	5.7 (0.6)	SS-16	9.5 (0.4)	SS-16
			MCI (*n* = 22)	9.6 (0.6)		7.1 (0.7)		9.0 (0.5)	
		Kim et al. (2020)	AD (*n* = 65)	4.69 (3.16)	YSK olfactory function test	1.1 (1.6)	YSK olfactory function test	4.4 (2.0)	YSK olfactory function test
			MCI (*n* = 26)	7.58 (4.03)		2.5 (1.6)		5.5 (1.9)	
		Peters et al. (2003)	AD (*n* = 14)	10.07 (2.3)	Common odors (16 items)	4.2 (1.7)	n-butanol	9.6 (2.3)	Triplet of pens
			MCI (*n* = 8)	10.5 (2.33)		5.4 (1.3)		9.5 (1.7)	
		Quarmley et al. (2017)	AD (*n* = 262)	7.82 (3.46)	SS-16	-	-	-	-
			MCI (*n* = 174)	9.94 (3.28)		-		-	
		Reijs et al. (2017)	AD (*n* = 42)	5.7 (2.4)	B-SIT	-	-	-	-
			MCI (*n* = 45)	6.7 (2.4)		-		-	
		Seligman et al. (2013)	AD (*n* = 172)	7.54 (3.53)	SS-16	-	-	-	-
			MCI (*n* = 112)	10.1 (3.39)		-		-	
		Steinbach et al. (2010)	AD (*n* = 30)	7.7 (3.3)	SS-16	4.5 (2.7)	n-butanol	8.8 (2.1)	Triplet of pens
			MCI (*n* = 29)	9.3 (4)		5.3 (2.8)		10.3 (2.6)	
		Tabert et al. (2005)	AD (*n* = 100)	23.72 (6.48)	UPSIT-40	-	-	-	-
			MCI (*n* = 147)	31.22 (3.1)		-		-	
		Umeda-Kameyama et al. (2017)	AD (*n* = 60)	3.6 (2.4)	OSIT-J	-	-	-	-
			MCI (*n* = 28)	5 (6.45)		-		-	
		Vasavada et al. (2017)	AD (*n* = 15)	15.5 (8.4)	UPSIT-40	-	-	-	-
			MCI (*n* = 21)	24.2 (8.6)		-		-	
		Wang et al. (2021)	AD (*n* = 52)	6.7 (2.3)	SS-16	-	-	-	-
			MCI (*n* = 129)	10.2 (2.5)		-		-	
		Ward et al. (2017)	AD (*n* = 13)	16.69 (6.51)	UPSIT-40	-	-	-	-
			MCI (*n* = 8)	21.63 (10.17)		-		-	
		Westervelt et al. (2008)	AD (*n* = 44)	6.5 (2.57)	B-SIT	-	-	-	-
			MCI (*n* = 88)	8.76 (2.59)				-	
		Woodward et al. (2017)	AD (*n* = 262)	18.76 (7.91)	UPSIT-40	-	-	-	-
			MCI (*n* = 110)	28.01 (7.97)		-		-	
		Woodward et al. (2018)	AD (*n* = 415)	19.36 (9.12)	UPSIT-40	-	-	-	-
			MCI (*n* = 192)	26.98 (8)		-		-	
		Wu et al. (2019)	AD (*n* = 37)	13.46 (6.09)	UPSIT-40	-	-	-	-
			MCI (*n* = 27)	19.11 (6.41)		-		-	
		Yoshii et al. (2019)	AD (*n* = 55)	3.5 (3)	OSIT-J	-	-	-	-
			MCI (*n* = 27)	7.2 (3.2)		-		-	
		Zhao et al. (2020)	AD (*n* = 88)	5.9 (2.7)	SS-16	-	-	-	-
			MCI (*n* = 87)	9.1 (2.7)		-		-	
LBD	OI (*n* = 4)	Forsberg et al. (2021)	AD (*n* = 83)	7 (2.7)	B-SIT	-	-	-	-
			LBD (*n* = 51)	4.5 (3.1)		-		-	
		Inagawa et al. (2020)	AD (*n* = 22)	4.4 (2.6)	OSIT-J	-	-	-	-
			LBD (*n* = 24)	2.5 (2.0)		-		-	
		Sato et al. (2011)	AD (*n* = 48)	4.1 (2.0)	The Open Essence Smell Identification test	-	-	-	-
			LBD (*n* = 38)	2.6 (1.9)		-		-	
		Westervelt et al. (2016)	AD (*n* = 60)	6.74 (2.64)	B-SIT	-	-	-	-
			LBD (*n* = 26)	4.12 (1.58)		-		-	
		Westervelt et al. (2003)	AD (*n* = 38)	7.68 (2.57)	B-SIT	-	-	-	-
			LBD (*n* = 138)	3.84 (1.89)		-			
		Williams et al. (2009)	AD (*n* = 27)	6.81 (3.1)	SS-16	-	-	-	-
			LBD (*n* = 21)	5 (2.3)		-		-	
		Yoo and Ye (2017)	AD (*n* = 244)	7.96 (2.49)	B-SIT	-	-	-	-
			LBD (*n* = 341)	7.11 (2.53)		-		-	
		Yoo et al. (2018)	AD (*n* = 237)	7.58 (2.56)	B-SIT	-	-	-	-
			LBD (*n* = 217)	6.66 (2.4)		-		-	
Depression	OI (*n* = 2)	Chen et al. (2018)	AD (*n* = 125)	5.8 (1.8)	SS-16	-	-	-	-
			Depression (*n* = 50)	9.9 (2.7)		-		-	
		Duff et al. (2002)	AD (*n* = 20)	0.4 (0.5)	PST	-	-	-	-
			Depression (*n* = 20)	2.7 (0.47)		-		-	
		McCaffrey et al. (2000)	AD (*n* = 20)	0.45 (0.6)	PST	-	-	-	-
			Depression (*n* = 20)	2.8 (0.41)		-		-	
		Pentzek et al. (2007)	AD (*n* = 20)	6.15 (2.18)	SS-16	-	-	-	-
			Depression (*n* = 20)	13.4 (1.35)		-		-	
		Solomon et al. (1998)	AD (*n* = 20)	0.8 (0.77)	PST	-	-	-	-
			Depression (*n* = 20)	2.8 (0.41)		-		-	
VaD	OI (*n* = 3)	Duff et al. (2002)	AD (*n* = 20)	18.38 (7.07)	UPSIT	-	-	-	-
			VaD (*n* = 20)	20.21 (7.11)		-		-	
		Motomura and Tomota (2006)	AD (*n* = 12)	4.5 (2.91)	SS-12	-	-		-
			VaD (*n* = 11)	6.2 (2.98)		-	-	-	
		Tkalčić et al. (2011)	AD (*n* = 15)	0.4 (0.5)	PST	-	-	-	-
			VaD (*n* = 11)	2.45 (0.89)		-		-	
PD	OI (*n* = 2)	Doty et al. (1991)	AD (*n* = 24)	3.5 (2.8)	UPSIT	-	-	-	-
			PD (*n* = 24)	6.8 (2.3)		-		-	
		Jesmanas et al. (2021)	AD (*n* = 20)	3.33 (3.16)	SOIT	-	-	-	-
			PD (*n* = 20)	6.09 (2.3)		-		-	
FTD	OI (*n* = 2)	Körtvélyessy et al. (2015)	AD (*n* = 27)	7.1 (2.9)	SS-12	-	-	-	-
			FTD (*n* = 16)	6.3 (2.4)		-		-	
		McLaughlin and Westervelt (2008)	AD (*n* = 14)	7.8 (2.7)	B-SIT	-	-	-	-
			FTD (*n* = 14)	7(3)		-		-	

SD, Standard deviation; OI, Olfactory identification; OT, Olfactory threshold; OD, Olfactory discrimination; AD, Alzheimer’s disease; MCI, mild cognitive impairment; LBD, Lewy body disease; VaD, vascular dementia; PD, Parkinson’s disease; FTD, frontotemporal dementia; -, data not informed.

### Meta-analysis results in comparison between AD and other conditions

There were 42 studies with sufficient information to calculate effect size in the meta-analysis (Doty et al., 1991; Solomon et al., 1998; McCaffrey et al., 2000; Duff et al., 2002; Peters et al., 2003; Westervelt et al., 2003; Tabert et al., 2005; Motomura and Tomota, 2006; Pentzek et al., 2007; Djordjevic et al., 2008; McLaughlin and Westervelt, 2008; Westervelt et al., 2008; Williams et al., 2009; Bahar-Fuchs et al., 2010; Steinbach et al., 2010; Bahar-Fuchs et al., 2011; Sato et al., 2011; Tkalčić et al., 2011; Seligman et al., 2013; Körtvélyessy et al., 2015; Westervelt et al., 2016; Quarmley et al., 2017; Reijs et al., 2017; Umeda-Kameyama et al., 2017; Vasavada et al., 2017; Ward et al., 2017; Woodward et al., 2017; Yoo and Ye, 2017; Chen et al., 2018; Doorduijn et al., 2018; Woodward et al., 2018; Yoo et al., 2018; Wu et al., 2019; Yoshii et al., 2019; Bathini et al., 2020; Forsberg et al., 2021; Inagawa et al., 2020; Kim et al., 2020; Zhao et al., 2020; Jesmanas et al., 2021; Wang et al., 2021; Chen et al., 2022). All studies except one compared AD with one specific disease by smell tests, while the remaining study tested AD patients, VaD patients, and individuals with depression simultaneously (Duff et al., 2002). The meta-analyses were conducted in comparison of AD with seven conditions separately, including MCI (*n* = 23), LBD (*n* = 8), depression (*n* = 5), VaD (*n* = 3), PD (*n* = 2), and FTD (*n* = 2). Most studies examined the olfactory identification function (Doty et al., 1991; Solomon et al., 1998; McCaffrey et al., 2000; Duff et al., 2002; Peters et al., 2003; Westervelt et al., 2003; Tabert et al., 2005; Motomura and Tomota, 2006; Pentzek et al., 2007; Djordjevic et al., 2008; McLaughlin and Westervelt, 2008; Westervelt et al., 2008; Williams et al., 2009; Bahar-Fuchs et al., 2010; Steinbach et al., 2010; Bahar-Fuchs et al., 2011; Sato et al., 2011; Tkalčić et al., 2011; Seligman et al., 2013; Körtvélyessy et al., 2015; Westervelt et al., 2016; Quarmley et al., 2017; Reijs et al., 2017; Umeda-Kameyama et al., 2017; Vasavada et al., 2017; Ward et al., 2017; Woodward et al., 2017; Yoo and Ye, 2017; Chen et al., 2018; Doorduijn et al., 2018; Woodward et al., 2018; Yoo et al., 2018; Wu et al., 2019; Yoshii et al., 2019; Bathini et al., 2020; Forsberg et al., 2021; Inagawa et al., 2020; Kim et al., 2020; Zhao et al., 2020; Jesmanas et al., 2021; Wang et al., 2021; Chen et al., 2022), while the olfactory threshold function was examined in the AD vs. MCI (Peters et al., 2003; Steinbach et al., 2010; Doorduijn et al., 2018; Kim et al., 2020), while the olfactory discrimination function was examined in the AD vs. MCI group (Peters et al., 2003; Djordjevic et al., 2008; Steinbach et al., 2010; Doorduijn et al., 2018; Kim et al., 2020). The five single studies excluded from our meta-analysis separately compared AD patients with elderly schizophrenia (Moberg et al., 1997), semantic dementia (Luzzi et al., 2007), corticobasal degeneration (Luzzi et al., 2007), unipolar major depression (Naudin et al., 2014), normal pressure hydrocephalus (Passler et al., 2017), and HIV-associated neurocognitive disorders (HAND; Sundermann et al., 2021) by smell tests. The study comparing unipolar major depression with AD (Naudin et al., 2014) was excluded since it was the only one that assessed long-term odor recognition memory capacity.

### AD vs. MCI

#### Olfactory identification

**Figure 2:**
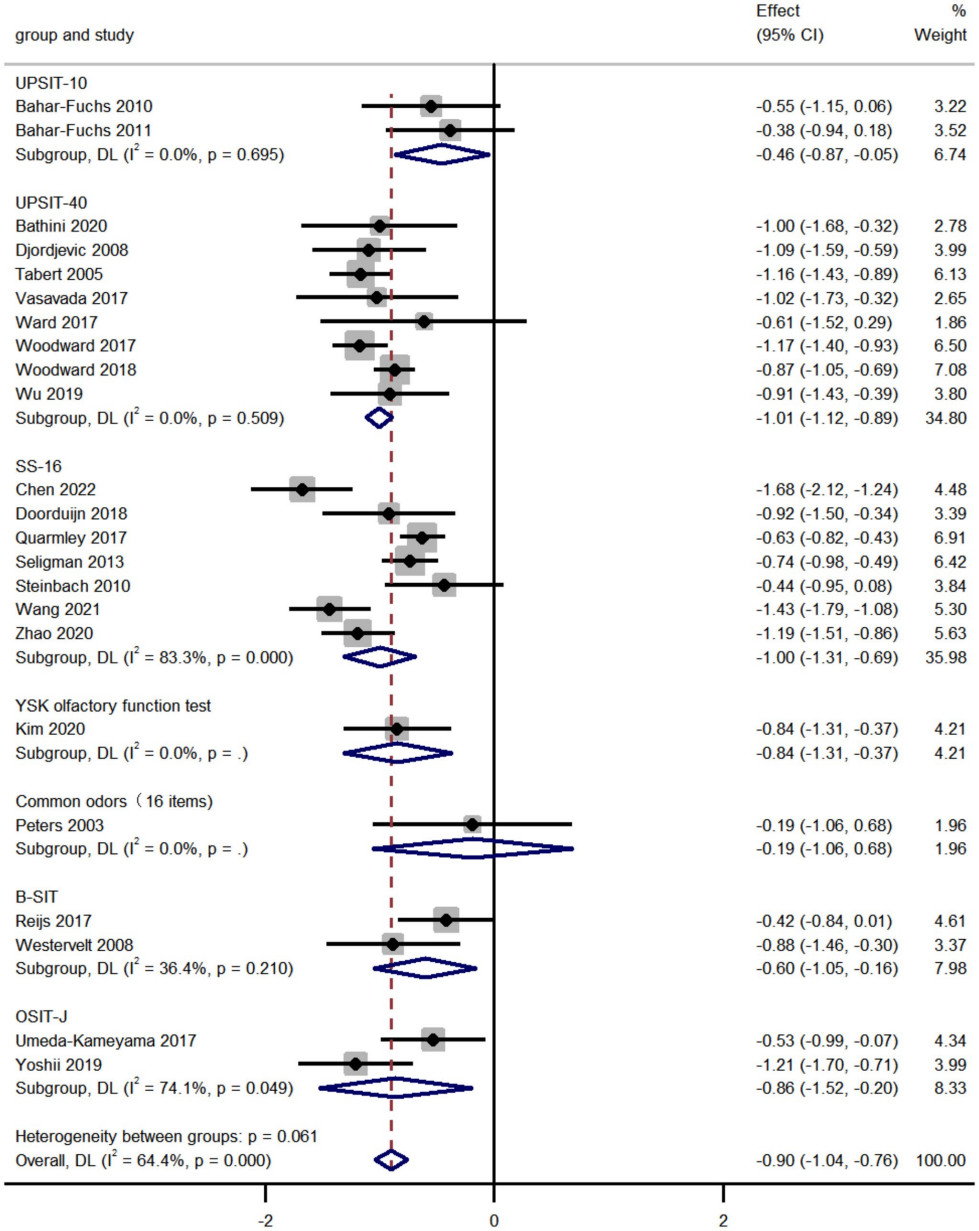
Forest plot of olfactory identification scores in AD and MCI.

We compared the olfactory identification scores in AD and MCI by analyzing the pooled data from 23 studies and found that the olfactory identification scores in AD were significantly lower than that in MCI (k = 23; d = −0.90; 95%CI −1.04 to −0.76; Figure 2; Peters et al., 2003; Tabert et al., 2005; Djordjevic et al., 2008; Westervelt et al., 2008; Bahar-Fuchs et al., 2010; Steinbach et al., 2010; Bahar-Fuchs et al., 2011; Seligman et al., 2013; Quarmley et al., 2017; Reijs et al., 2017; Umeda-Kameyama et al., 2017; Vasavada et al., 2017; Ward et al., 2017; Woodward et al., 2017; Doorduijn et al., 2018; Woodward et al., 2018; Wu et al., 2019; Yoshii et al., 2019; Bathini et al., 2020; Kim et al., 2020; Zhao et al., 2020; Wang et al., 2021; Chen et al., 2022). The results altered depending on the smell test used in various studies. High heterogeneity was found across studies (Q = 61.73; *P* < 0.0001; *I^2^* = 64.4%). No publication bias was found via Egger’s test (*p* = 0.804, Supplementary Figures 4, 5). The sensitivity analyses using a leave-one-out model showed no significant effect on results, indicating the overall pooled estimates were stable (95%CI −1.04 to −0.76, Supplementary Figure 6).

#### Olfactory threshold

**Figure 3:**
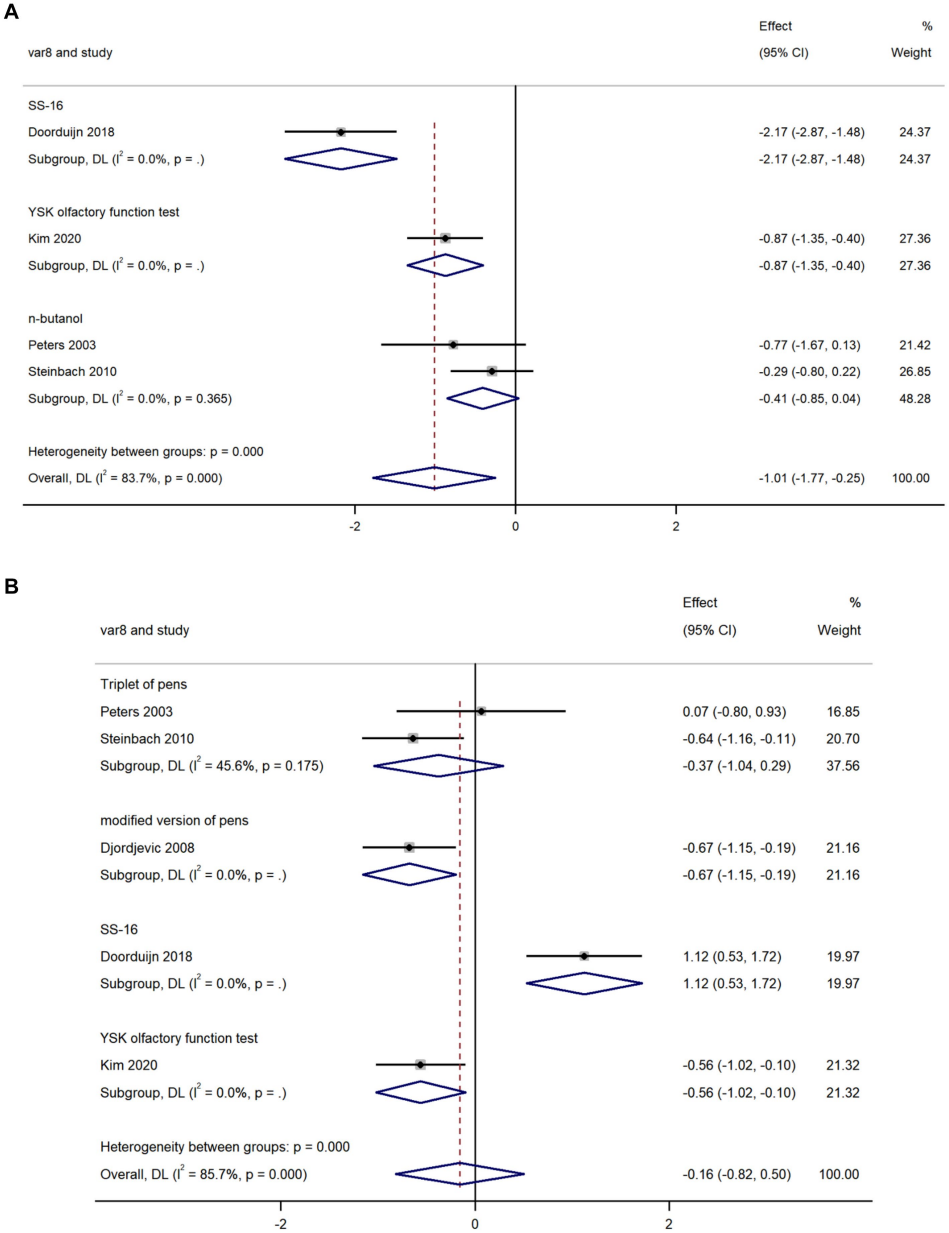
Forest plot of **(A)** olfactory threshold scores; and **(B)** olfactory discrimination scores in AD and MCI.

We used data from four studies to compare olfactory threshold scores in AD and MCI and found that olfactory threshold scores in AD were lower than that in MCI (k = 4; d = −1.01; 95%CI −1.77 to −0.25; Figure 3A; Peters et al., 2003; Steinbach et al., 2010; Doorduijn et al., 2018; Kim et al., 2020). High heterogeneity was found across studies (Q = 18.38; *P*<0.0001; *I^2^* = 83.7%).

#### Olfactory discrimination

We used data from five studies to compare olfactory discrimination scores in AD and MCI and revealed there was no significant difference in olfactory discrimination scores between the two groups (k = 5; d = −0.16; 95%CI −0.82–0.50; Figure 3B; Peters et al., 2003; Djordjevic et al., 2008; Steinbach et al., 2010; Doorduijn et al., 2018; Kim et al., 2020). High heterogeneity was found across studies (Q = 27.92; *P* < 0.0001; *I^2^* = 85.7%).

### AD vs. LBD

**Figure 4:**
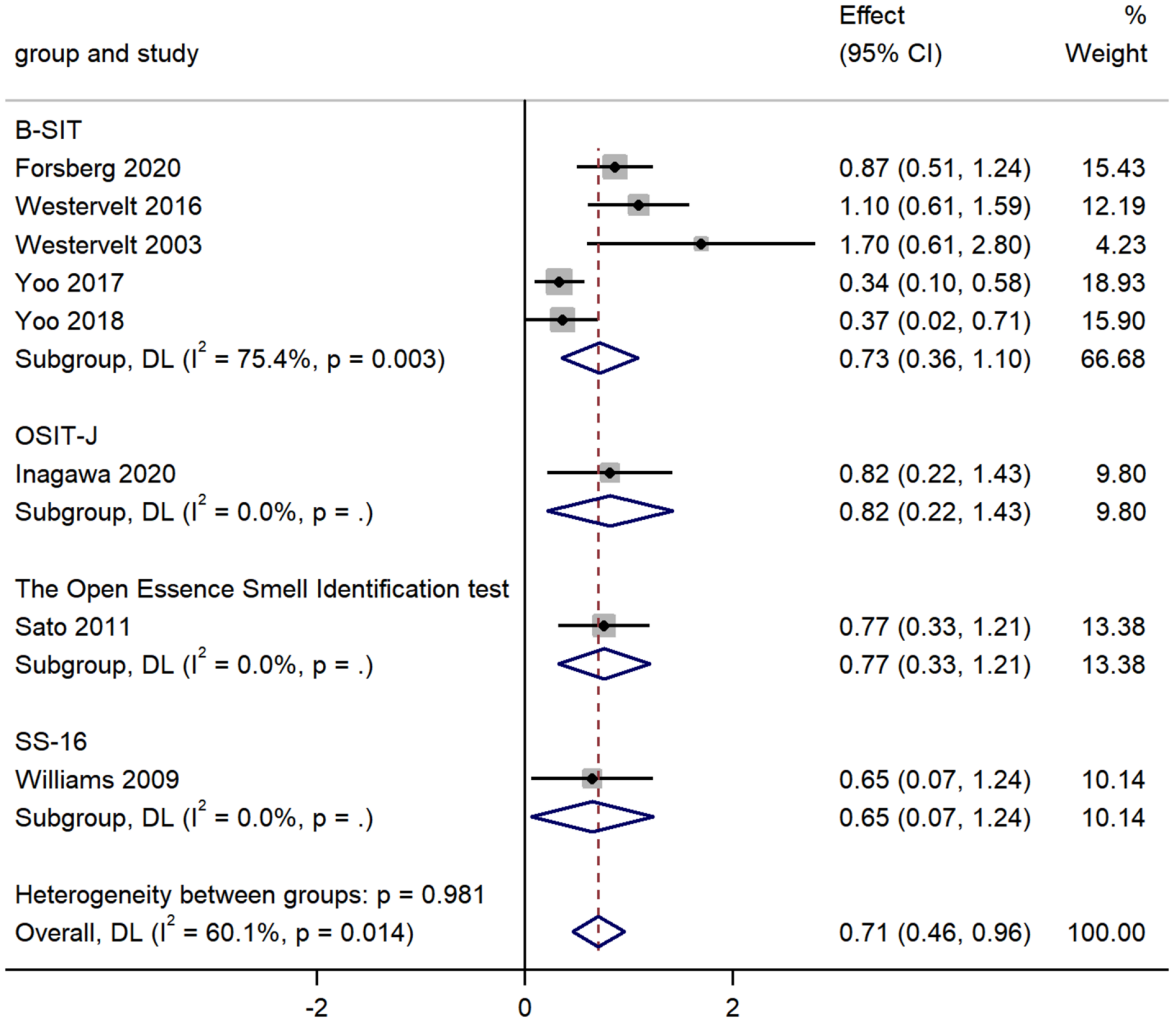
Forest plot of olfactory identification scores in AD and LBD.

The pooled data from eight studies comparing olfactory identification scores in AD and LBD patients revealed that the olfactory identification scores in AD were higher than those in LBD in all smell tests (k = 8; d = 0.71; 95%CI 0.46 to 0.96; Figure 4; Westervelt et al., 2003; Williams et al., 2009; Sato et al., 2011; Westervelt et al., 2016; Yoo and Ye, 2017; Yoo et al., 2018; Forsberg et al., 2021; Inagawa et al., 2020). We then found high heterogeneity across studies (Q = 17.54; *P* < 0.01; *I^2^* = 60.1%). The Egger’s test results indicated there was significant publication bias (*p* = 0.024; Supplementary Figures 7, 8) in the study, thus a trim-and-fill analysis was required to assess the stability of the combined results. The results revealed that after adding four studies, the effect value varied significantly (Supplementary Figure 9), which showed a unstable combined result.

The overall pooled estimates were stable (95%CI 0.45 to 0.96; Supplementary Figure 10), since there was no significant change in the total 95% CI and SMD while using a leave-one-out model for sensitivity analysis.

### AD vs. depression

**Figure 5:**
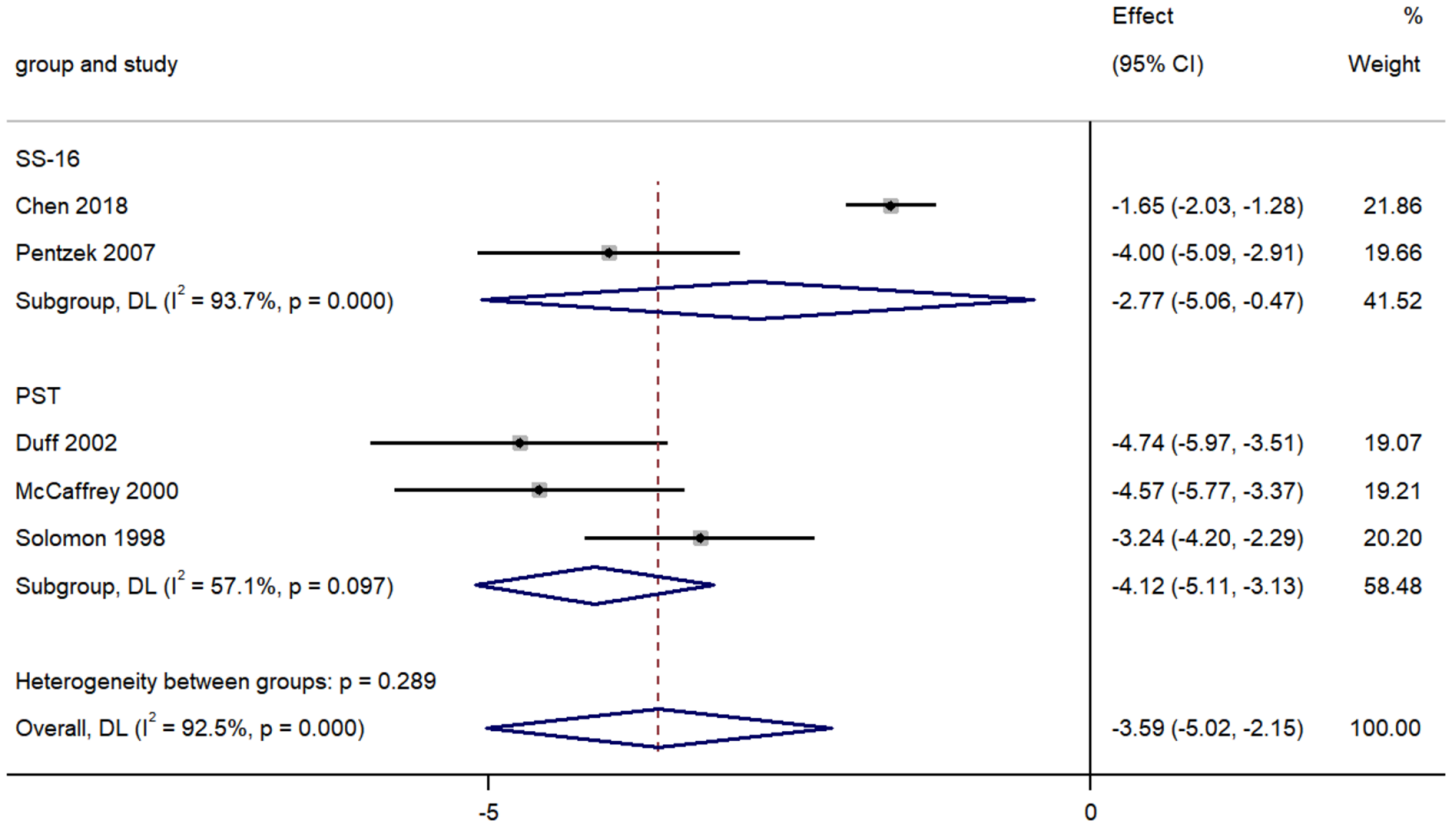
Forest plot of olfactory identification scores in AD and depression.

The pooled data from five studies comparing olfactory identification scores in individuals with AD and with depression revealed that the scores in AD patients were lower than those in depression in all tests (k = 5; d = −3.59; 95%CI −5.02 to −2.15; Figure 5; Solomon et al., 1998; McCaffrey et al., 2000; Duff et al., 2002; Pentzek et al., 2007; Chen et al., 2018). We then found there was high heterogeneity across studies (Q = 53.12; *P* < 0.0001; *I^2^* = 92.5%).

### AD vs. VaD

**Figure 6:**
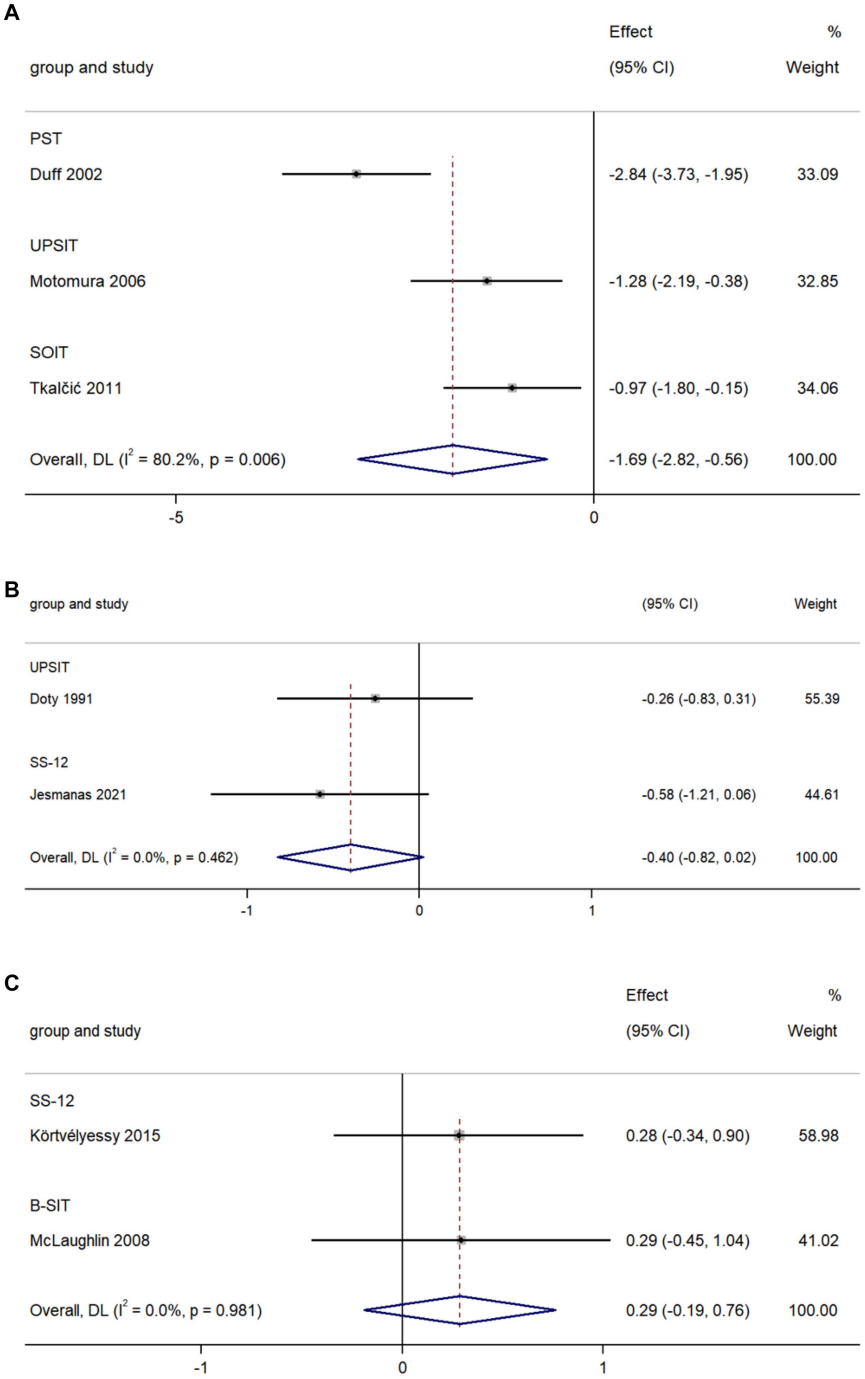
Forest plot of olfactory identification scores in AD and **(A)** VaD; **(B)** PD, and **(C)** FTD.

The pooled data from three studies comparing olfactory identification scores in AD and VaD patients indicated that the scores in AD patients were lower than those in VaD in all tests (k = 3; d = −1.96; 95%CI −2.82 to −0.56; Figure 6A; Duff et al., 2002; Motomura and Tomota, 2006; Tkalčić et al., 2011). High heterogeneity was found across studies (Q = 10.08; *p* = 0.006; *I^2^* = 80.2%).

### AD vs. PD

The pooled data from two studies comparing olfactory identification scores in AD patients and PD patients revealed a non-significant difference in these scores between the two groups in all tests (k = 2; d = −0.40; 95%CI −0.82 to 0.02; Figure 6B; Doty et al., 1991; Jesmanas et al., 2021). Low heterogeneity was found across studies (Q = 0.54; *p* = 0.46; *I^2^* < 0.01%).

### AD vs. FTD

The pooled data from two studies comparing olfactory identification scores in AD patients and FTD patients revealed there was no significant difference in olfactory identification scores between the two groups in all tests (k = 2; d = 0.29; 95%CI −0.19 to 0.76; Figure 6C; McLaughlin and Westervelt, 2008; Körtvélyessy et al., 2015). Low heterogeneity was found across studies (Q < 0.001; *p* = 0.981; *I^2^* < 0.01).

### Moderator analysis

#### Subgroup analysis

For olfactory identification, olfactory threshold, and olfactory discrimination, we performed subgroup analyses according to the methods of smell tests separately in AD vs. each disease. We found in group AD vs. MCI, there was significant heterogeneity in olfactory threshold (*p* < 0.001) and olfactory discrimination (*p* < 0.001), but not in olfactory identification (*p* = 0.061). No significant heterogeneity was found in other groups.

#### Meta-regression

Meta-regression was carried out to explore the sources of heterogeneity using demographic data, including age, sex (male%), MMSE scores, and years of education. Comparative studies in each meta-analysis with more than five articles were included. The summary of the meta-regression results was presented in Table 2. The statistical results indicated there was significant relationship between olfactory identification score and age (*p* = 0.031), between the score and MMSE (*p* = 0.016) as well as between the score and education years (*p* = 0.023) in group AD vs. LBD. No significant association was found between any other variables and odor identification scores. Further details were presented in Supplementary Figures 11–18.

**Table 2:** Statistical results of meta-regression analyses in groups AD vs. MCI or LBD.

Disease		K	T	95%CI	Adj R^2^	*p-*value
AD vs. MCI	Age	23	1.54	−0.01 to 0.07	13.38%	0.138
	Sex (male%)	21	1.50	−0.01 to 0.03	3.37%	0.150
	MMSE	16	0.78	−0.04 to 0.09	−6.08%	0.451
	Education	14	1.28	−0.04 to 0.14	4.25%	0.224
AD vs. LBD	Age	7	2.97	0.01 to 0.12	100.00%	0.031^*^
	Sex (male%)	8	−0.23	−0.04 to 0.03	−36.15%	0.823
	MMSE	8	−3.31	−0.36 to −0.05	100.00%	0.016^*^
	Education	6	3.60	0.05 to 0.37	100.00%	0.023^*^

K, number of the studies included in the meta-regression analyses; Adj R^2^, adjusted R^2^; MMSE, Mini-mental state examination; AD, Alzheimer’s disease; MCI, Mild cognitive impairment; LBD, Lewy body disease.

* *P* < 0.05.

## Discussion

The meta-analysis began with a thorough search for the most updated available data on comparison of olfactory function between AD and MCI, also between AD and other neurological diseases. We then qualitatively and quantitatively analyzed the olfactory performance between AD and various conditions, and further tested the potential effect of smell test methods on results through subgroup analyses. Finally, we performed meta-regression analyses to explore the sources of heterogeneity and the potential moderating factors on the included smell tests.

The meta-analysis revealed significant disparity between AD and MCI, LBD, depression, and VaD in olfactory functions. Most of above smell tests evaluated on olfactory identification, and olfactory threshold were applied in a few studies to distinguishing AD from MCI. Meanwhile, it was suggested that neither PD nor FTD could be differentiated from AD by different smell tests. Nevertheless, this irrelevance may be due to small sample sizes, and large confidence intervals and few types of smell tests in meta-analyses. Therefore, more studies, larger sample sizes, as well as more methods of smell tests are required in the future to draw more accurate results.

In the meantime, olfactory impairment was reported in AD patients, PD patients, and MCI patients, respectively, in prior meta-analyses. Comparing AD and PD patients to health controls, a large effect size (*d* = 3.36) was demonstrated between AD patients and health controls in Mesholam’s meta-analysis, in which patients with AD and PD showed a severe decline in olfactory identification (Mesholam et al., 1998). Another research by Rahayel et al. which characterized AD and PD patterns of deficits across different olfactory tasks also observed significant impairment in olfaction in AD patients compared to health controls with a large effect size (*d* = 1.73; Rahayel et al., 2012). While there was a much smaller degree of olfactory impairment in MCI patients (Roalf et al., 2017). Also, in line with Mesholam’s meta-analysis, no differences in olfactory deficits were found between patients with AD and PD in our study. This may suggest that AD and PD had a similar disturbance in olfactory identification (*p* = 0.46).

### AD vs. MCI

We expanded and updated synthesized data on the olfactory function of AD patients and MCI patients. Consistent with another study directly comparing olfactory function in AD patients and MCI patients (Jung et al., 2019), our results revealed that the olfactory impairment of AD patients was significantly worse than that of MCI patients with a large effect size since the Cohen’s d was −0.90, while a medium-to-large effect size (*d* = 0.64) was reported in that previous meta-analysis. We also noticed that depending on the methods of smell test, the differences of olfactory impairment between AD patients and MCI patients varied as well.

We applied subgroup analyses of smell test methods in data processing. It is notable that the UPSIT (40 items) was the most preferred smell test in comparison of AD patients and MCI patients. Among all smell tests, the UPSIT fared best in differentiating the two, with scores significantly lower in AD patients than that in MCI patients. The UPSIT, also known as a starch and sniff test, is a multi-optional smell test that encourages self-administration (Doty et al., 1984). Multiple versions of the UPSIT according to cultural adaptation have been published and now it is a widely used and highly credible smell identification test worldwide. However, not all smell tests can distinguish the olfactory performance of AD patients from MCI patients. As shown in the forest plot Figure 2, the common odors (16 items) failed in differentiating AD patients from MCI patients. Our forest plot showed that in addition to the UPSIT, the SS was also commonly used in identifying olfactory function in MCI and AD. According to the number of odorants, there are three versions of the SS, including SS-12, SS-16, and SS-48, while the SS-16 is most frequently used in the studies included in the meta-analysis. The SS also covers three aspects of olfactory functions, olfactory identification, olfactory discrimination, and olfactory threshold (Hummel et al., 1997).

The use of meta-regression is another novel feature of our meta-analysis. Through it, we found none of the variables we included (age, sex, MMSE scores, education years) had significant association with olfactory identification scores in group AD vs. MCI.

The insignificant association between MMSE scores and olfactory identification scores is somewhat surprising because the recent claim supported that olfactory identification requires a fully functional peripheral chemosensory system and central processing including memory and cognition (Pacyna et al., 2022). Some studies have indicated a significant link between olfactory identification impairment and cognition decline (Devanand, 2016; Wang et al., 2021). Using a mixed model of continuous cognitive outcomes in a longitudinal study of geriatric cohorts, researchers discovered that impaired olfaction was associated with incidence of MCI and amnestic mild cognitive impairment (aMCI) as well as worsened cognitive performance during follow-up (Roberts et al., 2016). Based on the facts mentioned above, it makes common sense that the MMSE, a screening method frequently used in AD to grade the cognitive status (Folstein et al., 1975), should be associated with the difference of olfactory identification scores between AD and MCI patients. However, our result is consistent with a meta-analysis by Roalf et al. which challenged the prior understanding since the authors found no significant relationship between MMSE scores and olfactory scores in comparing MCI patients and healthy controls (Roalf et al., 2017). The insignificant association may be due to the MMSE test is less informative than other detecting methods. The Montreal cognitive assessment, which is more sensitive than MMSE test, shows a higher advantage in distinguishing between MCI and AD, which also confirms that MMSE may be too simple to effectively distinguish between these two conditions (Pinto et al., 2019). The MoCA test contains more robust measures of higher-level language abilities as well as visuospatial and executive function (Nasreddine et al., 2005). Therefore, we appeal for a more complete and sensitive cognitive assessment, such as the MoCA test, to evaluate cognitive impairment in people with AD spectrum disorders.

### AD vs. LBD

Additionally, we contrasted the results of olfactory identification tests between AD and LBD, the latter comprising dementia with Lewy bodies (DLB) and Parkinson’s disease dementia. While DLB is the second most prevalent form of neurodegenerative dementia (McKeith et al., 2017). The clinical course of LBD typically progresses rapidly, which also calls for an effective tool for early screening and differential diagnosis. For the above reasons, it is an urgent need for a straightforward, user-friendly, and affordable test in diagnosis of LBD. Our meta-analysis showed that LBD has more severe olfactory impairment in olfactory identification than that in AD, with a somewhat larger effect size (*d* = 0.71). Consistently, several studies highlighted the advantages of smell tests in the detection and screening of AD and LBD, and the olfactory identification test appears to be a reliable approach and could match the need for early differential diagnosis of the above two diseases (Williams et al., 2009; Yoo et al., 2018). Since AD pathology is frequent accompanied with LBD at autopsy, atypical LBD patients (combined with AD pathology) can appear clinically equivalent to patients with AD, while their primary pathology can later be discovered in autopsy (McKeith et al., 2016; Robinson et al., 2018; Walker et al., 2021). Whereas our results revealed different degree of olfactory impairment in AD and LBD patients, the further application of smell test in the future may be helpful for clinical identification of these patients with atypical syndrome.

Furthermore, the subgroup analysis in comparison of AD with LBD revealed that unlike the group AD vs. MCI, B-SIT was the most commonly used smell test in olfactory identification. B-SIT is a condensed cross-cultural version of the UPSIT with only 12 items instead of 40 items in UPSIT (Doty et al., 1996). The widespread application of B-SIT in patients with dementia may be attributed to some reasons as followed. Firstly, the patients with dementia needed assistance from others and their short-term memory window is relatively small, so the smell test for them should be conveniently carried out at the bedside and as quick as possible (Kjelvik et al., 2007). Moreover, B-SIT’s efficacy has gradually been demonstrated in various cultures and numerous neurodegenerative illnesses, suggesting that in the future it may be applied more frequently in dementia patients (Cao et al., 2019). Lastly, the diagnostic effectiveness of B-SIT was no less than that of other methods, since there were little differences between the various smell test methods (B-SIT, the Open Essence Smell Identification test, OSIT-J, SS-16) in the meta-analysis.

Another intriguing result we observed in the moderator analysis was that the years of education was an influential factor in meta-analysis and the difference of olfactory identification scores between LBD and AD patients was shown to be positively correlated with the years of education. The result somehow challenged the previous view that longer years of education were associated with lower risk of dementia (Vemuri et al., 2014). But a few studies revealed similar results to us. One indicated that longer years of education were more prevalent in DLB individuals than in patients with AD (Boot et al., 2013). Furthermore, it was found in another study that longer years of education was associated with earlier LBD onset (Schaffert et al., 2020). This phenomenon is not limited to LBD since a case–control study also showed that subjects with higher education and physicians had an increased risk of PD (Frigerio et al., 2005). Further investigation is needed to explore this contradictory phenomenon. Age and MMSE scores may also account for the heterogeneity across studies in group AD vs. LBD, which is consistent with previous opinion that olfactory function declines with age (Doty, 2018), and that higher MMSE scores imply better cognitive status and may represent diseases at earlier stage, which may be the reason that there is a smaller olfactory score difference between LBD and AD patients.

### AD vs. depression

Depression is a potential risk factor of Alzheimer’s disease (Walker et al., 2021). Additionally, depressive symptom is considered to be the most significant non-cognitive clinical manifestation of AD (Aalten et al., 2007). Given the description above, it is evident that depression and AD are easily confused. Therefore, the assessment of clinically relevant biomarkers, such as smell tests, may be effective and convenient methods for early screening and identification of depression and AD.

The value of smell tests in discrimination of these two diseases has already been verified both in previous studies and our meta-analysis (Solomon et al., 1998; McCaffrey et al., 2000; Duff et al., 2002; Pentzek et al., 2007; Chen et al., 2018). Our results revealed that the olfactory identification ability of AD patients was substantially worse than that of depression patients, with a sizeable effect size (*d* = −3.59). While the patients with depression usually maintained a relatively intact olfactory identification function. Through subgroup analysis, we discovered that the PST was most frequently administrated in comparative studies to assess the olfactory function of AD and depression and with a better discernment. The PST, a simplified version of the UPSIT, is a 3-item microencapsulated “scratch-and-sniff” test (Duff et al., 2002).

### Strength and weakness

This is the first comprehensive systematic review and meta-analysis comparing olfactory impairment in AD and other neurodegenerative conditions. Our study revealed that some neurodegenerative conditions, including MCI, LBD, depression, and VaD could be distinguished from AD by smell tests. We also analyzed various methods of smell tests and conducted subgroup analyses to evaluate if they can different AD from MCI and from other neurodegenerative conditions. Ultimately, we discovered that the UPSIT and its cross-cultural simplified version, the B-SIT, were the most commonly used smell tests. Additionally, we performed moderator analyses to investigate the potential impact of several demographic factors on the outcomes of the smell test. Overall, we discovered that the smell test is an appropriate as well as practical clinical technique for early detection and discrimination of AD and other neurodegenerative disorders.

However, there are some limitations in our meta-analysis. Firstly, sample sizes in the comparative meta-analysis of AD with several other diseases were relatively small. Only five studies were included in the comparison of olfactory impairment between AD and depression, three studies were included to compare AD with VaD, while only two studies were included to compare AD with PD or with FTD. The insufficient number of research may affect the validity of these analyses and made it infeasible to analyze sensitivity, publication bias as well as moderator variables in these groups. As the literature updates, these above statistical analyses should be supplemented in the future. In addition, we only included literature in English since there was no eligible Chinese literature matched with our inclusion criteria in searching Chinese article database. Furthermore, due to the dearth of data, we were unable to perform moderator analyses on several variables, such as duration years and smoking history. Furthermore, due to the insufficient data, we were unable to test the sensitivity or specificity of smell tests to assess the power of each smell test in clinical discrimination, and this should be improved in subsequent research as well. Finally, the literature we reviewed was restricted to cross-sectional research, while further longitudinal researches need to be proceeded in the future to explore how olfactory impairment in AD and other conditions progress over time.

## Conclusion

In summary, our finding suggest that smell tests have potential capacity in distinguishing AD from MCI, and from other diseases, while it can also be widely used as a dynamic monitoring biomarker suitable for research and clinical use for its low cost and ease of testing. As the application of the UPSIT is currently frequent, we also recommend more use of the B-SIT in the future since it is easy to perform with various cross-cultural forms. Eventually, it is worth mentioning that, although the smell test can be applied for early screening as well as the differential diagnosis of AD and other conditions, we do not recommend to use smell tests to diagnose these conditions independently. For neurodegenerative conditions, early smell tests may be helpful in identifying patients who need further expensive or invasive pathological diagnostic tests and patients who need to take preventative measures due to possible cognitive loss.

## Data availability statement

The original contributions presented in the study are included in the article/Supplementary material, further inquiries can be directed to the corresponding author.

## Author contributions

HP designed the study and revised and edited the manuscript. SL, ZJ, JZ, ZL, RL, and YQ performed the literature search, data extraction, and analysis. SL, ZJ, and JZ wrote the manuscript. All authors contributed to the article and approved the submitted version.

## Funding

This work was supported by Grants from Natural Science Foundation of Guangdong Province (grant no. 2023A1515010268), Key Areas Research and Development Programs of Guangdong Province (2022B1111020006), and Guangdong Provincial Science and Technology Plan Project (grant no. 2013A022100035).

## Conflict of interest

The authors declare that the research was conducted in the absence of any commercial or financial relationships that could be construed as a potential conflict of interest.

## Publisher’s note

All claims expressed in this article are solely those of the authors and do not necessarily represent those of their affiliated organizations, or those of the publisher, the editors and the reviewers. Any product that may be evaluated in this article, or claim that may be made by its manufacturer, is not guaranteed or endorsed by the publisher.
