# Supplementary material for: Disparity of smell tests in Alzheimer’s disease and other neurodegenerative disorders: a systematic review and meta-analysis
Source: Front Aging Neurosci. 2023 Sep 7;15:1249512. doi: 10.3389/fnagi.2023.1249512 (PMC10512741; doi:10.3389/fnagi.2023.1249512)
Supplement: Supplementary file 1 [file Data_Sheet_1.docx]

**Supplementary table 1:** *characteristics of the included studies*

| Diseases | References |  | Age  Years (SD) | Sex  Male (%) | MMSE(SD) | Diagnostic criterion | Education  Years (SD) |
| --- | --- | --- | --- | --- | --- | --- | --- |
| MCI | Bahar-Fuchs 2010 | AD(n=20) | 73.4(8.5) | 55 | 22.9(3.2) | NINCDS-ADRDA | 14.8(5.3) |
|  |  | aMCI(n=24) | 74.2(9.5) | 66 | 25.9(2.5) | Perterson, aMCI | 12.1(3.4) |
|  | Bahar-Fuchs 2011 | AD(n=25) | 73.0(8.5) | 48 | - | NINCDS-ADRDA | 14.1(5.8) |
|  |  | aMCI(n=25) | 74.4(9.3) | 64 | - | Winblad, aMCI | 13.6(4.1) |
|  | Bathini 2020 | AD(n=17) | 71.1(6.6) | 35 | 14.2(4.3) | the clinical protocol CERVD 2016-01627 | - |
|  |  | MCI(n=21) | 73.2(8.1) | 38 | 22.5(1.5) | the clinical protocol CERVD 2016-01628 | - |
|  | Chen 2022 | AD(n=31) | 69.9（11.0） | 39 | - | NINCDS-ADRDA | 8.2（4.6） |
|  |  | MCI(n=118) | 67.9（7.7） | 29 | - | Perterson, MCI | 9.1（3.7） |
|  | Djordjevic 2008 | AD(n=27) | 77.0（55-88) | 52 | 22.85 | NINCDS-ADRDA | 12.8 |
|  |  | aMCI(n=51) | 75.4(59-86) | 49 | 27.26 | Perterson, aMCI | 11.5 |
|  | Doorduijn 2018 | AD(n=30) | 70(9) | 47 | 24.0(3) | Albert, Alzhimer | - |
|  |  | MCI(n=22) | 70(7) | 72 | 26.0(1) | - | - |
|  | Kim 2020 | AD(n=65) | 78.3(7.1) | 25 | 12.3(5.0) | - | - |
|  |  | MCI(n=26) | 75.0(9.6) | 27 | 21.9(4.3) | - | - |
|  | Peters 2003 | AD(n=14) | 72.2(5.7) | - | 23.2(3.1） | NINCDS-ADRDA | - |
|  |  | aMCI(n=8) | 72.5(5.0) | - | 28.8(1.4) | Perterson, aMCI | - |
|  | Quarmley 2017 | AD(n=262) | 75.2(8.2) | 37 | - | NINCDS-ADRDA | 14.1(3.8) |
|  |  | MCI(n=174) | 72.5(8.6) | 47 | - | Pertersen, MCI | 15.0(3.6) |
|  | Reijs 2017 | AD(n=42) | 66.6(8.4) | 50 | 23.2(3.2) | NINCDS-ADRDA | 11.3(3.9) |
|  |  | MCI(n=45) | 69.5(9.4) | 56 | 27.2(1.8) | Pertersen, MCI | 10.5(3.6) |
|  | Seligman 2013 | AD(n=172) | 76.0(7.5) | 36 | - | - | 14.1(3.7) |
|  |  | MCI(n=112) | 72.6(8.2) | 39 | - | - | 15.0(4.1) |
|  | Steinbach 2010 | AD(n=30) | 73.3(7.8) | 43 | 21.7(4.7) | NINCDS-ADRDA | - |
|  |  | MCI(n=29) | 71.7(7.7) | 59 | 26.8(2.2) | Pertersen, MCI | - |
|  | Tabert 2005 | AD(n=100) | 71.7(9.5) | 46 | 20.0(6.0) | NINCDS-ADRDA | 13.1(4.4) |
|  |  | MCI(n=147) | 67.4(9.9) | 45 | 27.3(3.3) | Tabert, MCI | 15.0(4.3) |
|  | Umeda-Kameyama 2017 | AD(n=60) | 80.5(6.5) | 32 | 21.0(3.3) | - | - |
|  |  | aMCI(n=28) | 81.0(6.0) | 36 | 24.7(2.7) | - | - |
|  | Vasavada 2017 | AD(n=15) | 71.9(11.9) | 33 | 18.9(5.4) | NINCDS-ADRDA | - |
|  |  | MCI(n=21) | 73.2(9.0) | 48 | 26.5(1.9) | Perterson, MCI | - |
|  | Wang 2021 | AD(n=52) | 71.2(10.3) | 44 | 11.7(5.1) | NINCDS-ADRDA | - |
|  |  | MCI(n=129) | 67.8(8.6) | 32 | 24.9(3.0) | Perterson, MCI | - |
|  | Ward 2017 | AD(n=13) | 76.8(7.0) | 31 | - | NINCDS-ADRDA | 17.0(2.8) |
|  |  | MCI(n=8) | 76.1(6.3) | 63 | - | Albert, MCI | 15.5(3.5) |
|  | Westervelt 2008 | AD(n=44) | 76.3(5.4) | - | 23.8(3.1) | NINCDS-ADRDA | 12.2(2.8) |
|  |  | MCI(n=88) | 74.6(8.2) | - | 27.0(2.3) | Pertersen, aMCI | 13.0(3.3) |
|  | Woodward 2017 | AD(n=262) | 75.6(8.3) | 50 | 22.8(3.7) | NINCDS-ADRDA | 15.0(2.8) |
|  |  | MCI(n=110) | 74.1(9.0) | 52 | 27.6(2.1) | Pertersen, aMCI | 15.0(2.7) |
|  | Woodward 2018 | AD(n=415) | 74.6(8.5) | 48 | 22.8(4.3) | NINCDS-ADRDA | 14.1(4.4) |
|  |  | MCI(n=192) | 73.2(9.1) | 54 | 27.2(3.5) | Pertersen, aMCI | 13.7(5.5) |
|  | Wu 2019 | AD(n=37) | 66.9(10.3) | 46 | - | NINCDS-ADRDA | 6.8(5.3) |
|  |  | MCI(n=27) | 68.0(7.6) | 48 | - | Pertersen, MCI | 8.5(5.5) |
|  | Yoshii 2019 | AD(n=55) | 80.0(7.0) | 38 | - | DSM-IV | - |
|  |  | MCI(n=27) | 76.0(10.0) | 30 | - | Morris, aMCI | - |
|  | Zhao 2020 | AD(n=88) | 67.7(4.2) | 53 | 17.0(2.1) | NIA-AA | 10.4(2.5) |
|  |  | MCI(n=87) | 66.2(4.3) | 46 | 25.7(1.4) | Pertersen, MCI | 10.5(2.6) |
| LBD | Forsberg 2020 | AD(n=83) | - | 47 | 21.5(4.9) | - | - |
|  |  | LBD(n=51) | - | 84 | 23.3(4.3) | - | - |
|  | Inagawa 2020 | AD(n=22) | 80.5(range,61-88) | 50 | 20.4(4.8) | NINCDS-ADRDA | 12.1(2.8) |
|  |  | LBD(n=24) | 82.4(range,73-91) | 58 | 22.9(3.4) | McKeith, DLB | 13.1(2.8) |
|  | Sato 2011 | AD(n=48) | 76.2(6.8) | 31 | 21.9(1.7) | NINCDS-ADRDA | 12.2(3.2) |
|  |  | LBD(n=38) | 78.1(6.5) | 47 | 22.6(2.8) | McKeith, DLB | 12.1(2.9) |
|  | Westervelt 2016 | AD(n=60) | 77.5(7.1) | 32 | 23.4(3.4) | NINCDS-ADRDA | 12.5(3.3) |
|  |  | LBD(n=26) | 77.6(7.3) | 47 | 23.1(4.0) | McKeith, DLB | 13.0(3.3) |
|  | Westervelt 2003 | AD(n=38) | 77.3(8.8) | 33 | 20.7(3.8) | NINCDS-ADRDA | 11.4（2.6） |
|  |  | LBD(n=138) | 77.0(11.7) | 22 | 22.6(6.2) | McKeith, DLB | 13.6（4.3） |
|  | Williams 2009 | AD(n=27) | 77.7（8.4） | 52 | 22.6(2.8) | NINCDS-ADRDA | - |
|  |  | LBD(n=21） | 80.0(5.8） | 48 | 24.1(2.5) | McKeith, DLB | - |
|  | Yoo 2017 | AD(n=244) | 71.3(8.7) | 37 | 24.4(4.3) | - | 9.9(4.8) |
|  |  | LBD(n=341) | 70.5(8.2) | 42 | 25.3(3.8) | - | 9.1(5.1) |
|  | Yoo 2018( | AD(n=237) | 71.9(8.9) | 36 | 23.6(4.6) | NINCDS-ADRDA | 10.2(5.0) |
|  |  | LBD(n=217) | 70.2(8.4) | 45 | 24.9(4.1) | McKeith, DLB | 9.1(5.0) |
| Depression | Chen 2018 | AD(n=125) | 71.9(9.9) | 44 | 12.4(5.1) | NINCDS-ADRDA | 6.8(4.1) |
|  |  | Depression(n=50) | 66.7(6.2) | 23 | 22.7(5.3) | DSM-IV | 8.0(5.0) |
|  | Duff 2002 | AD(n=20) | 73.9(8.9) | 30 | 20.1(6.2) | DSM-IV | 11.8(3.7) |
|  |  | Depression(n=20) | 71.4(5.4) | 40 | 28.8(1.4) | DSM-IV | 12.7(4.8) |
|  | McCaffrey 2000 | AD(n=20) | 74.2(7.9) | 35 | 20.9(5.2) | DSM-IV | 13.4(3.6) |
|  |  | Depression(n=20) | 67.6(7.3) | 45 | 28.6(1.5) | DSM-IV | 13.9(3.5) |
|  | Pentzek 2007 | AD(n=20) | 76.0(9.1) | 25 | - | NINCDS-ADRDA | 10.5(3.2) |
|  |  | Depression(n=20) | 73.5(5.6) | 25 | - | ICD-10 | 10.7(2.2) |
|  | Solomon 1998 | AD(n=20) | 74.5(7.8) | 40 | - | DSM-IV | - |
|  |  | Depression(n=20) | 69.4(7.7) | 35 | - | DSM-IV | - |
| VaD | Duff 2002 | AD(n=20) | 73.9(8.9) | 30 | 20.1(6.2) | DSM-IV | 11.8(3.7) |
|  |  | VaD(n=20) | 74.4(6.5) | 25 | 23.1(5.0) | DSM-IV | 12.7(4.8) |
|  | Motomura 2006 | AD(n=12) | 69.0(3.8) | - | 12.5(2.8) | NINCDS-ADRDA | 9.2(2.3) |
|  |  | VaD(n=11) | 71.0(6.5) | - | 11.7(2.9) | DSM-IV | 8.5(1.5) |
|  | Tkalčić 2011 | AD(n=15) | - | 27 | - | CAMDEX | - |
|  |  | VaD(n=11) | - | 27 | - | CAMDEX | - |
| PD | Doty 1991 | AD(n=24) | 68.7(8.5) | 42 | - | Doty, Alzheimer | - |
|  |  | PD(n=24) | 61.0(7.7) | 42 | - | Doty, Parkingson | - |
|  | Jesmanas 2021 | AD(n=20) | 72.3(8.7) | 55 | 17.6(5.4) | NINCDS-ADRDA | 13.0(3.7) |
|  |  | PD(n=20) | 64.1(10.1) | 55 | 28.5(1.6) | Hughes, Parkingson | 14.7(2.9) |
| FTD | Körtvélyessy 2015 | AD(n=27) | 72.7(6.9) | 33 | 21.9(4.0) | NINCDS-ADRDA | - |
|  |  | FTD(n=16) | 68.4(7.7) | 31 | 19.6(5.7) | Neary, FTD | - |
|  | McLaughlin 2008 | AD(n=14) | 68.8(8.9) | 43 | 24.1(3.0) | NINCDS-ADRDA | 13.1(2.9) |
|  |  | FTD(n=14) | 64.9(10.0) | 43 | 20.7(5.1) | Neary, FTD | 12.8(3.1) |

MMSE: Mini-mental state examination.

**Supplementary table 2:**  *Summery of quality appraisal for each study.*

| **Study** | **RISK OF BIAS** | | | | **APPLICABILITY CONCERNS** | | |
| --- | --- | --- | --- | --- | --- | --- | --- |
|  | **PATIENT SELECTION** | **INDEX TEST** | **REFERENCE STANDARD** | **FLOW AND TIMING** | **PATIENT SELECTION** | **INDEX TEST** | **REFERENCE STANDARD** |
| Bahar-Fuchs et al. 2010 | ☹ | ☹ | ☺ | ? | ☺ | ☺ | ☺ |
| Bahar-Fuchs et al. 2011 | ☹ | ☹ | ☺ | ☹ | ☺ | ☺ | ☺ |
| Bathini et al. 2020 | ☹ | ☹ | ☺ | ☺ | ☺ | ☺ | ☺ |
| Chen et al. 2018 | ☹ | ☹ | ☺ | ☺ | ☺ | ☺ | ☺ |
| Chen et al. 2022 | ☹ | ☹ | ☺ | ☺ | ☺ | ☺ | ☺ |
| Djordjevic et al. 2008 | ☹ | ☹ | ☺ | ☺ | ☺ | ☺ | ☺ |
| Doorduijn et al. 2018 | ☹ | ☹ | ☺ | ☺ | ☺ | ☺ | ☺ |
| Doorduijn et al.2020 |  | ☹ | ☺ | ☺ | ☺ | ☺ | ☺ |
| Doty et al. 1991 | ☹ | ☹ | ☺ | ☺ | ☺ | ☺ | ☺ |
| Duff et al. 2002 | ☹ | ☹ | ☺ | ? | ☺ | ☺ | ☺ |
| Forsberg et al. 2020 | ☹ | ☹ | ☺ | ? | ☺ | ☺ | ☺ |
| Gilbert et al. 2004 | ☺ | ☹ | ☺ | ? | ☺ | ☺ | ☺ |
| Inagawa et al. 2020 | ☹ | ☹ | ? | ? | ☺ | ☺ | ☺ |
| Jesmanas et al. 2021 | ☹ | ☹ | ☺ | ? | ☺ | ☺ | ☺ |
| Kim et al. 2020 | ☹ | ☹ | ? | ☹ | ☺ | ☺ | ☺ |
| Körtvélyessy et al. 2015 | ☹ | ☹ | ☺ | ? | ☺ | ☺ | ☺ |
| Luzzi et al. 2007 | ☹ | ☹ | ☺ | ☺ | ☺ | ☺ | ☺ |
| McCaffrey et al. 2000 | ☹ | ☹ | ☺ | ☺ | ☺ | ☺ | ☺ |
| McLaughlin et al. 2008 | ☹ | ☹ | ☺ | ☹ | ☺ | ☺ | ☺ |
| Motomura et al. 2006 | ☹ | ☹ | ☺ | ? | ☺ | ☺ | ☺ |
| Olichney et al. 2005 | ☺ | ☹ | ☺ | ☺ | ☺ | ☺ | ☺ |
| Pentzek et al. 2007 | ☹ | ☹ | ☺ | ☺ | ☺ | ☺ | ☺ |
| Peters et al. 2003 | ☹ | ☹ | ☺ | ☺ | ☺ | ☺ | ☺ |
| Quarmley et al. 2017 | ☹ | ☹ | ☺ | ☺ | ☺ | ☺ | ☺ |
| Reijs et al. 2017 | ☹ | ☹ | ? | ☺ | ☺ | ☺ | ☺ |
| Sato et al. 2011 | ☹ | ☹ | ☺ | ☺ | ☺ | ☺ | ☺ |
| Seligman et al. 2013 | ☹ | ☹ | ☺ | ☺ | ☺ | ☺ | ☺ |
| Solomon et al. 1998 | ☹ | ☹ | ☺ | ☺ | ☺ | ☺ | ☺ |
| Steinbach et al. 2010 | ☹ | ☹ | ☺ | ☺ | ☺ | ☺ | ☺ |
| Tabert et al. 2005 | ☹ | ☹ | ☺ | ☺ | ☺ | ☺ | ☺ |
| Tkalčić et al. 2011 | ☹ | ☹ | ? | ☺ | ☺ | ☺ | ☺ |
| Umeda-Kameyama et al. 2017 | ☹ | ☹ | ? | ? | ☺ | ☺ | ☺ |
| Vasavada et al. 2017 | ☹ | ☹ | ☺ | ? | ☺ | ☺ | ☺ |
| Wang et al. 2021 | ☹ | ☹ | ☺ | ☺ | ☺ | ☺ | ☺ |
| Ward et al. 2017 | ☹ | ☹ | ? | ? | ☺ | ☺ | ☺ |
| Westervelt et al. 2003 | ☹ | ☹ | ☺ | ☺ | ☺ | ☺ | ☺ |
| Westervelt et al. 2008 | ☹ | ☹ | ☺ | ? | ☺ | ☺ | ☺ |
| Westervelt et al. 2016 | ☹ | ☹ | ☺ | ☺ | ☺ | ☺ | ☺ |
| Williams et al. 2009 | ☹ | ☹ | ☺ | ☺ | ☺ | ☺ | ☺ |
| Woodward et al. 2017 | ☹ | ☹ | ☺ | ☹ | ☺ | ☺ | ☺ |
| Woodward et al. 2018 | ☹ | ☹ | ☺ | ☹ | ☺ | ☺ | ☺ |
| Wu et al. 2019 | ☹ | ☹ | ☺ | ☺ | ☺ | ☺ | ☺ |
| Yoo et al. 2017 | ☹ | ☹ | ☺ | ? | ☺ | ☺ | ☺ |
| Yoo et al. 2018 | ☹ | ☹ | ☺ | ☹ | ☺ | ☺ | ☺ |
| Yoshii et al. 2019 | ☹ | ☹ | ☺ | ☺ | ☺ | ☺ | ☺ |
| Zhao et al. 2020 | ☹ | ☹ | ☺ | ☺ | ☺ | ☺ | ☺ |

☺Low Risk ☹High Risk? Unclear Risk


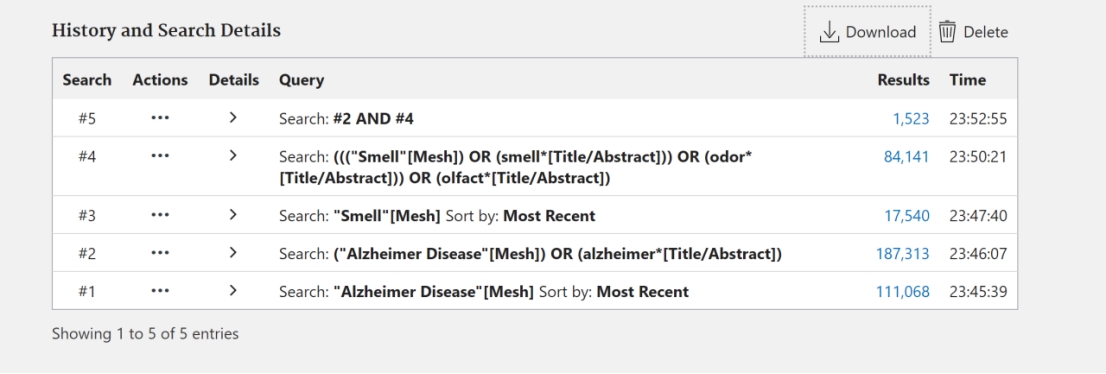


**Supplementary figure 1:**  *Search results and details in PubMed*


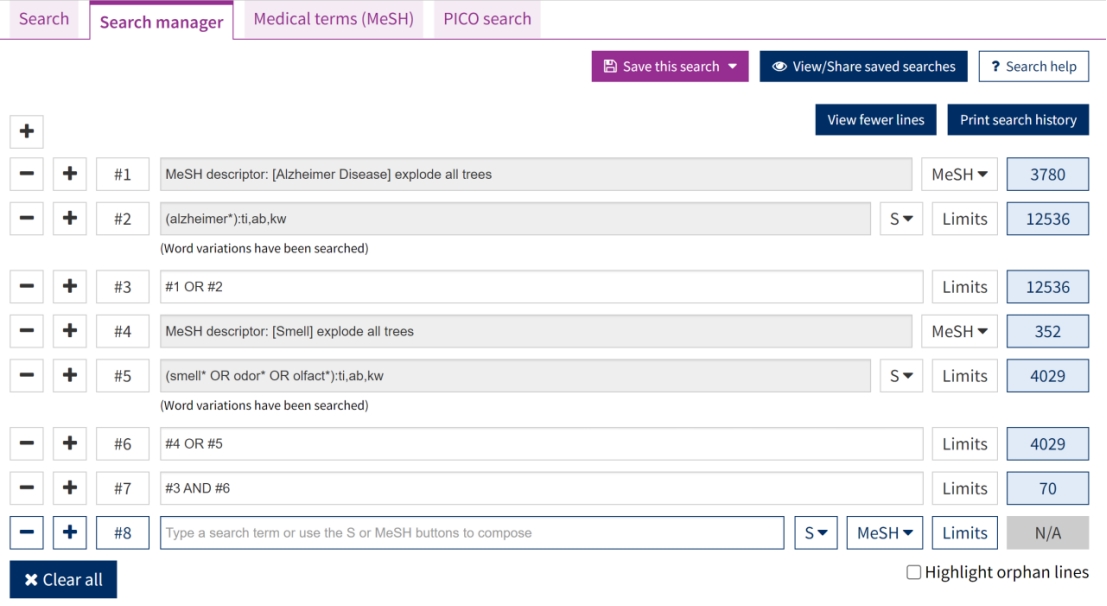


**Supplementary figure 2:**  *Search results and details in Cochrane*


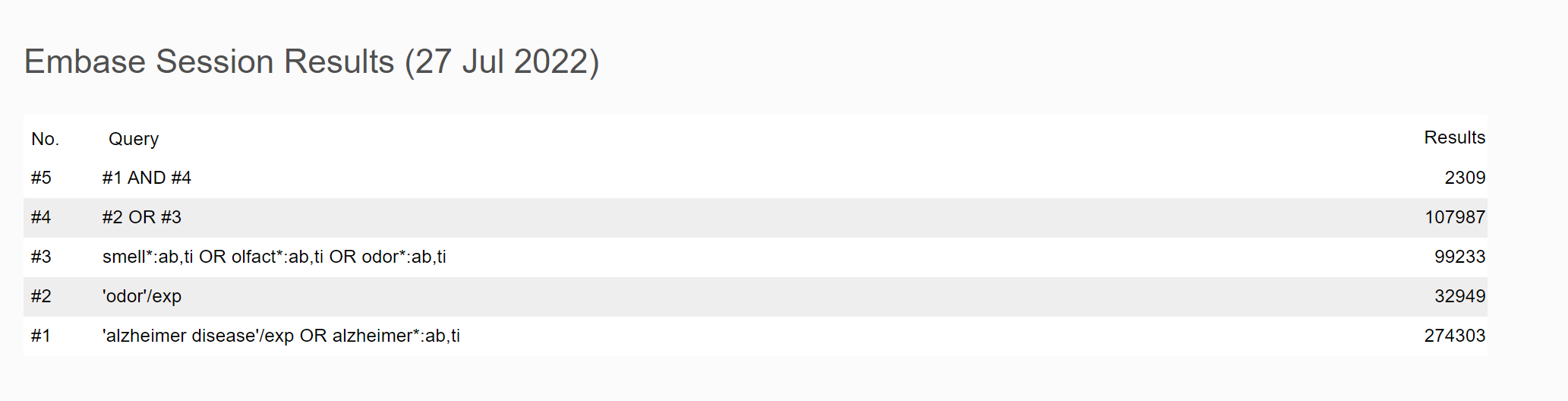


**Supplementary figure 3:**  *Search results and details in Embase*


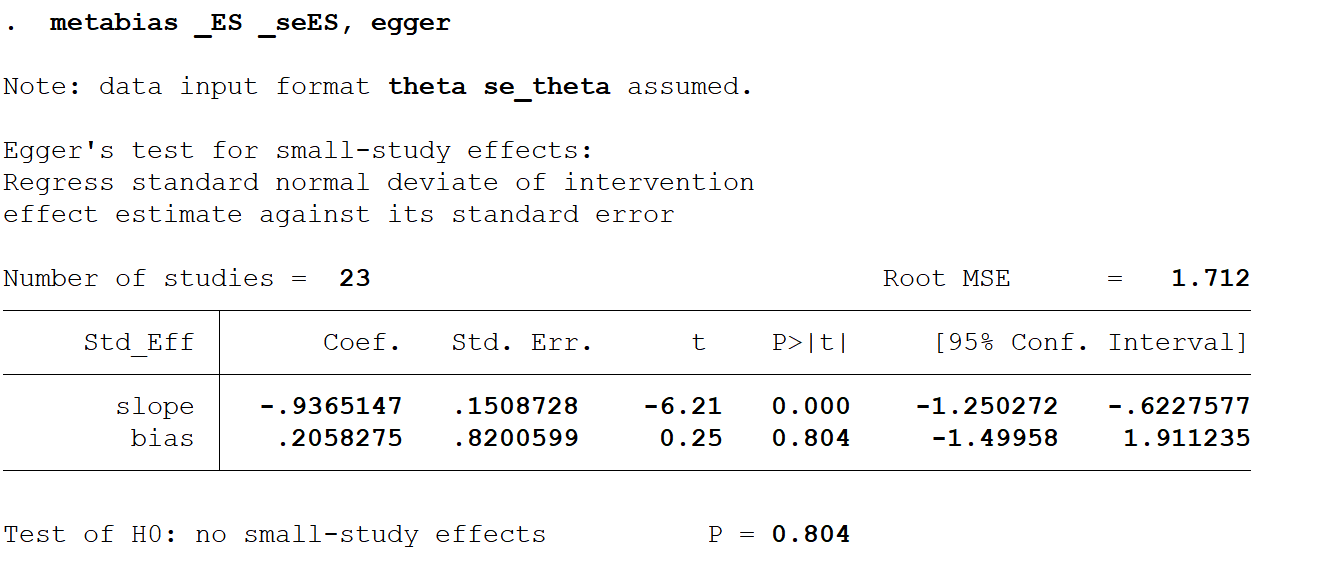


**Supplementary figure 4:** *publication bias in AD versus mild cognitive impairment* （p＞0.05）


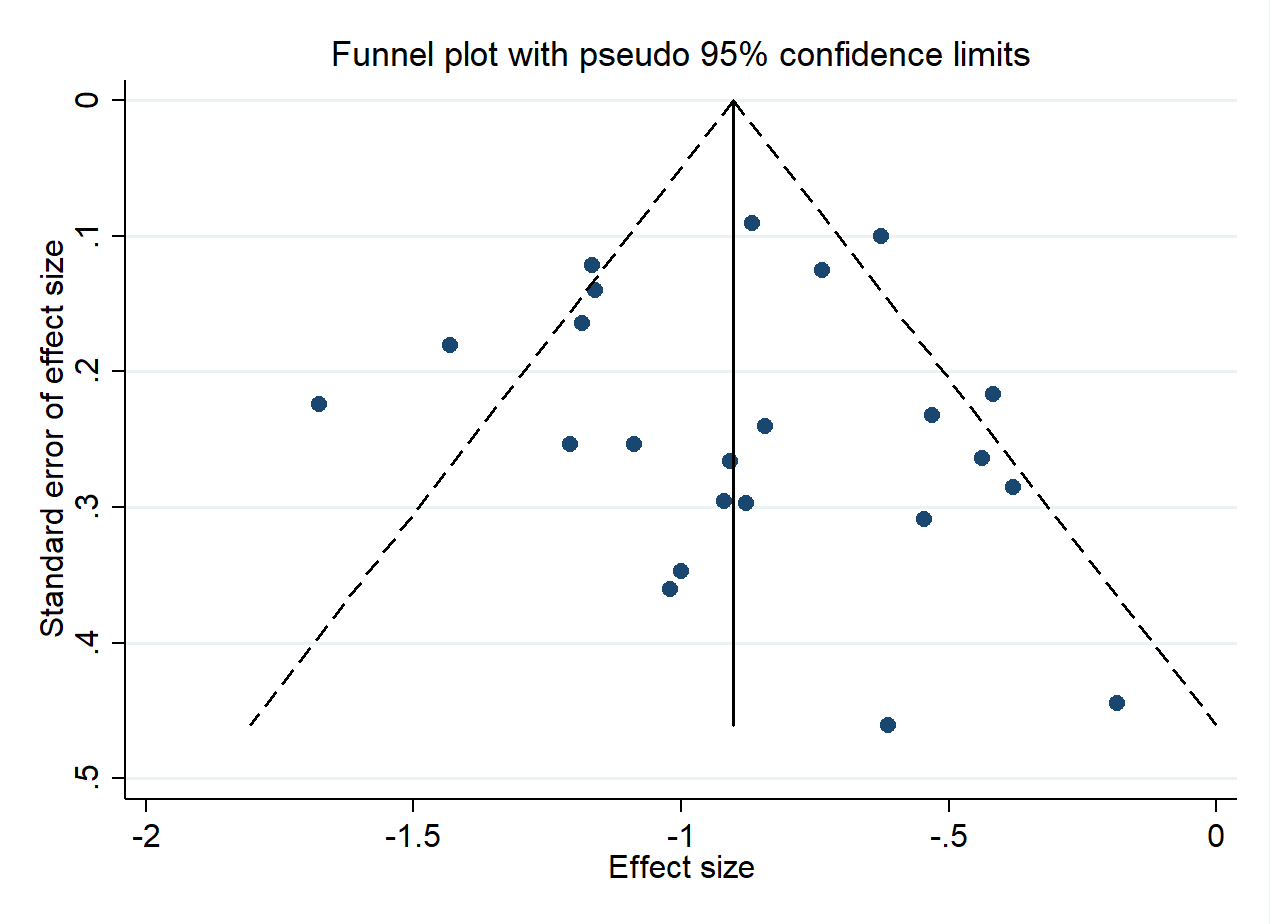


**Supplementary figure 5:** *funnel plot of the publication bias of olfactory identification scores in AD versus MCI.*


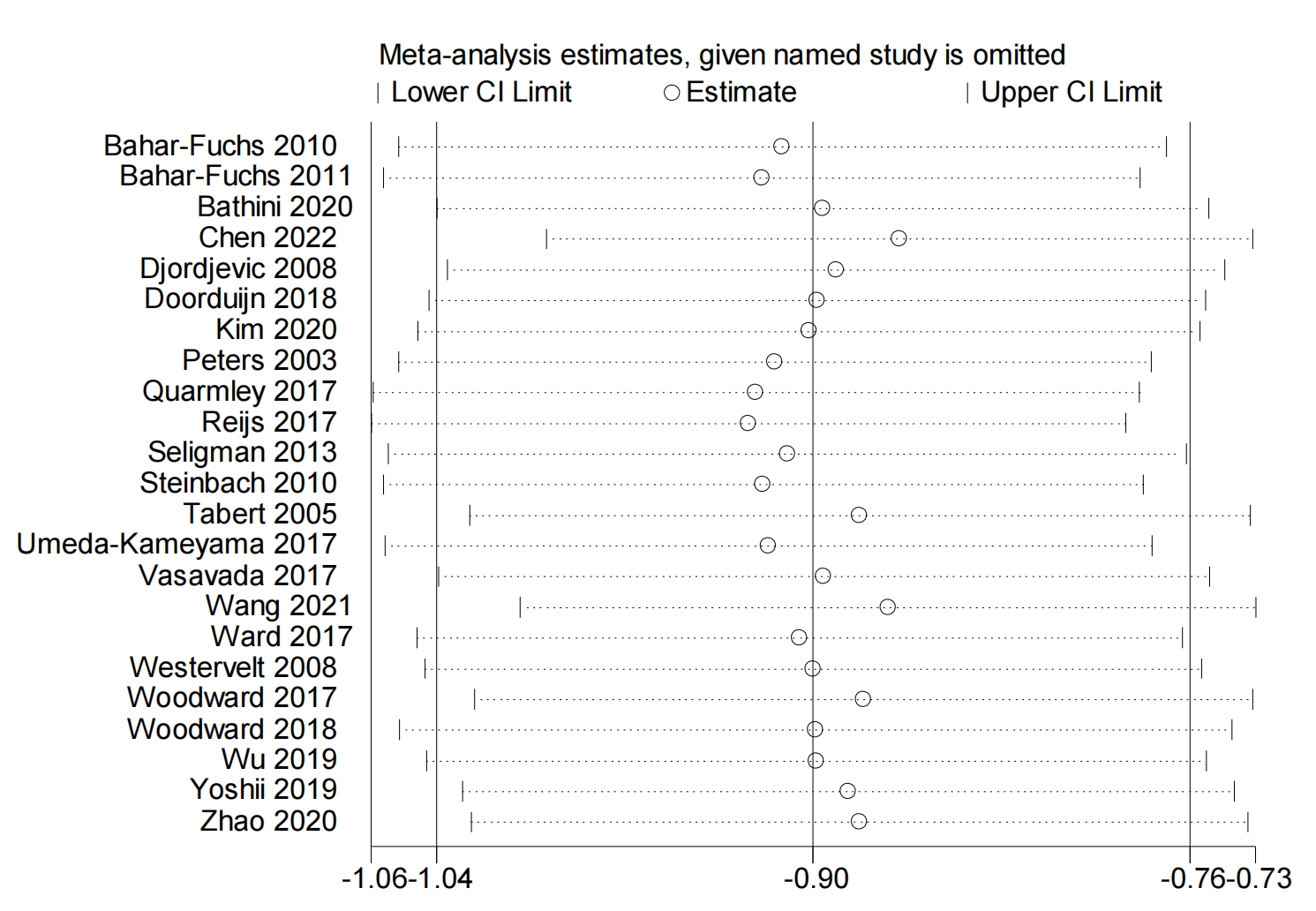


**Supplementary figure 6:** *sensitivity analysis results in AD versus mild cognitive impairment*


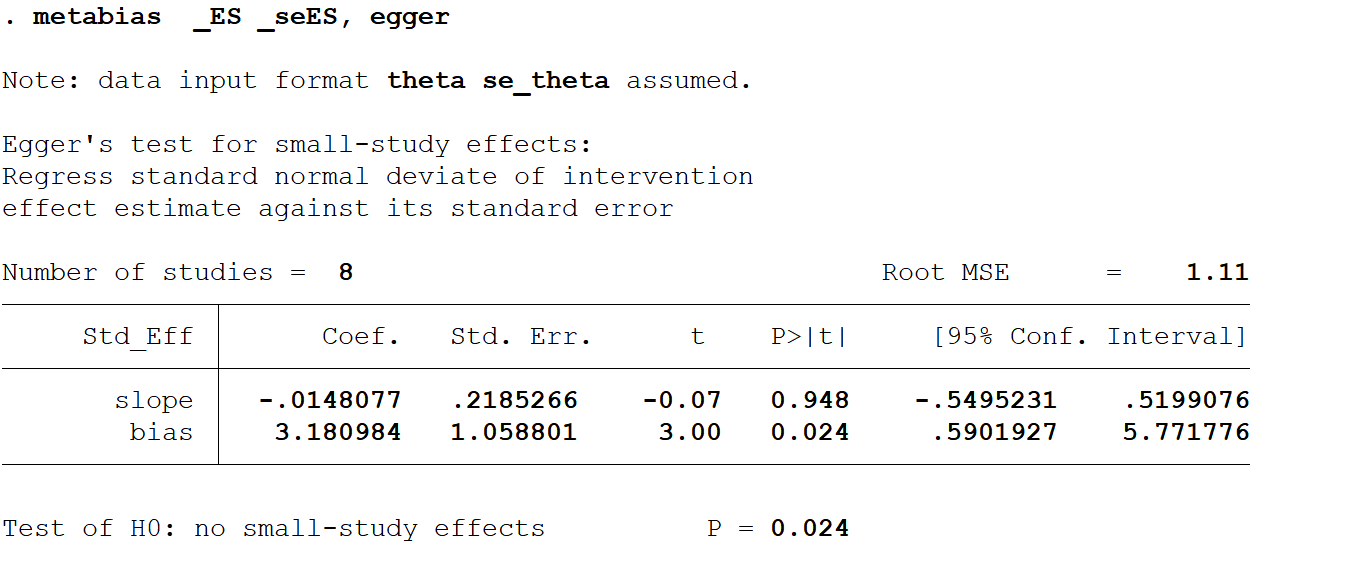
**supplementary figure 7:** *Publication bias in AD versus Lewy body disease*

(p=0.024)


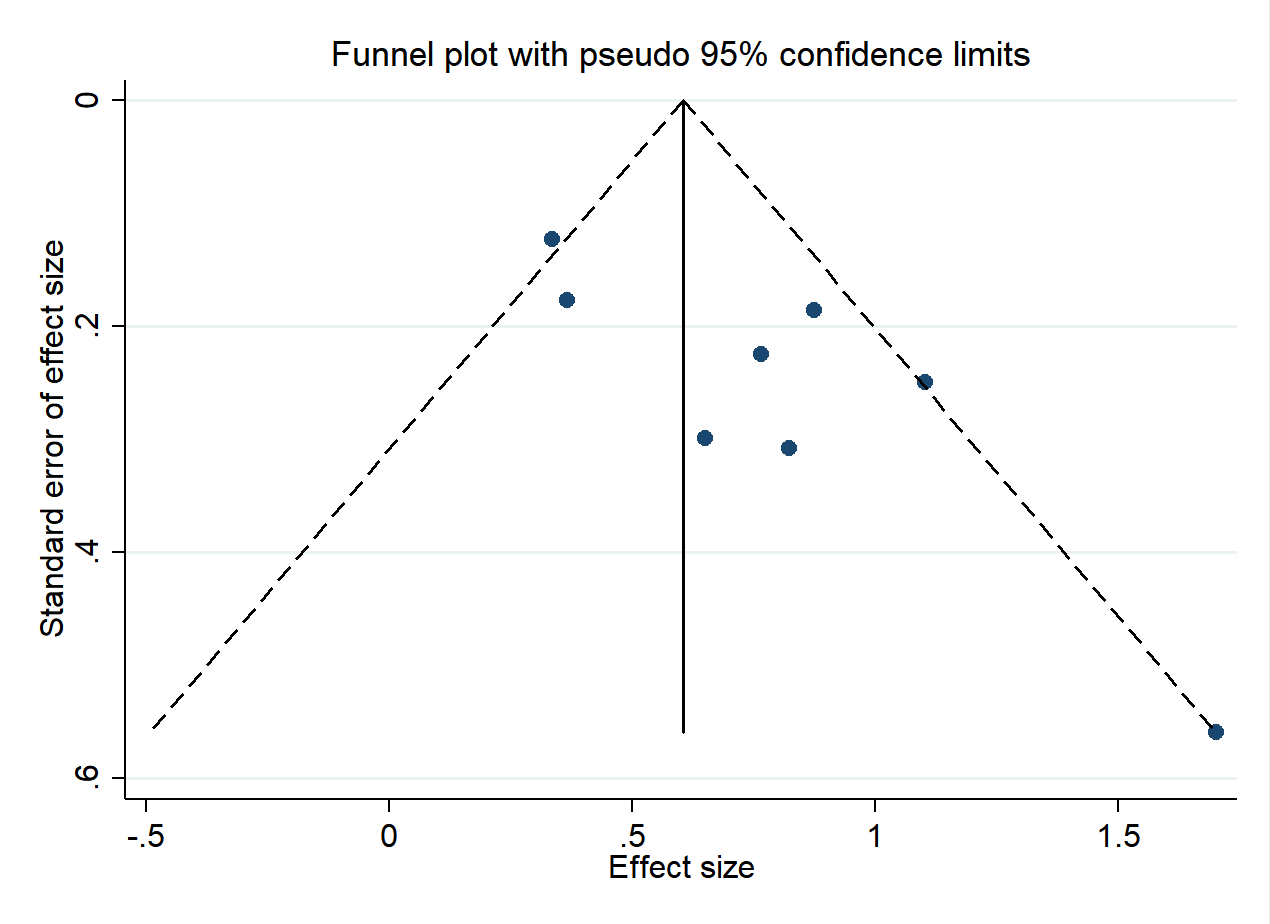


**Supplementary figure 8**: *funnel plot of the publication bias of olfactory identification scores in AD versus LBD.*


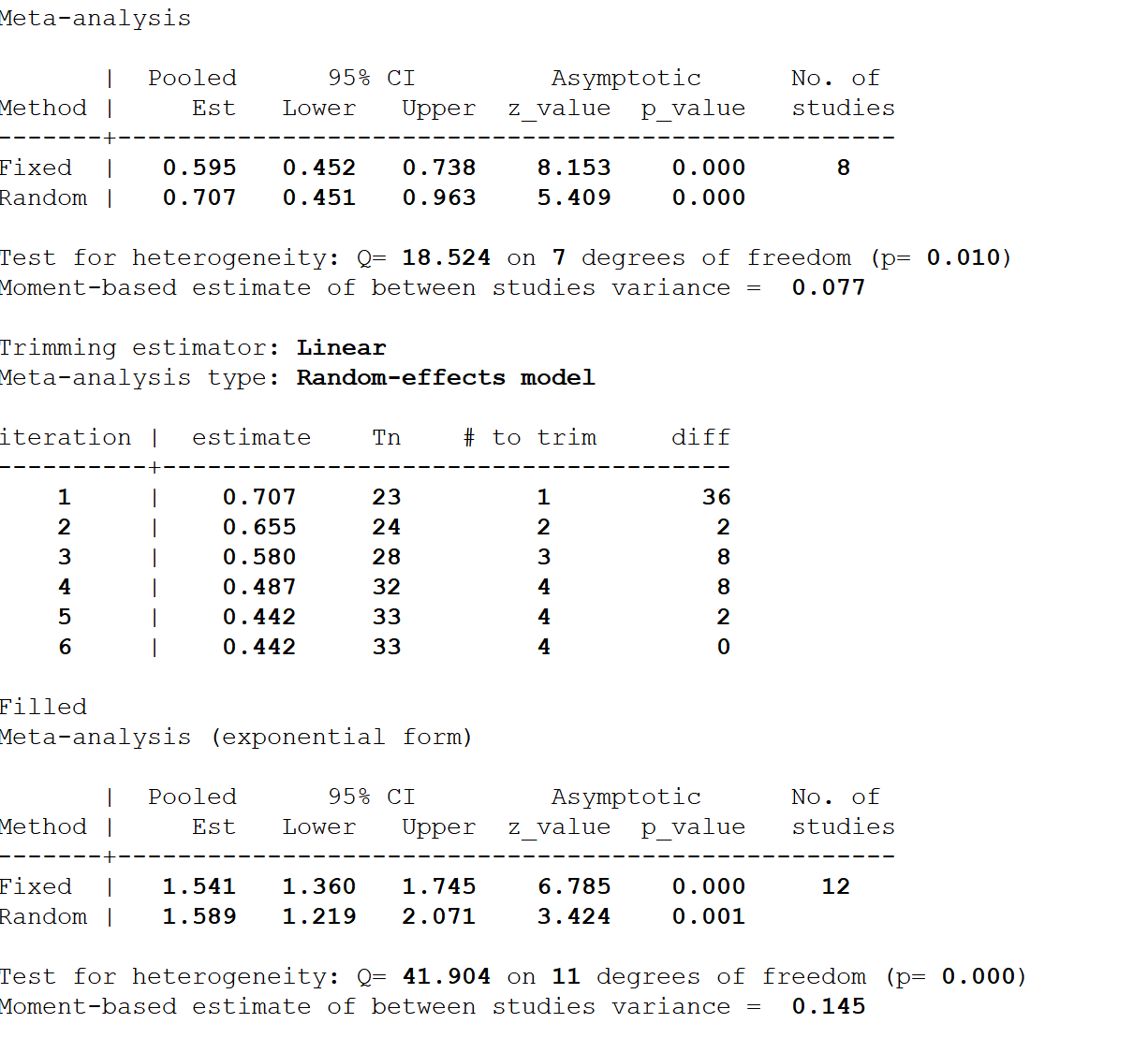


**Supplementary figure 9:** *Trim-and-Fill analysis results in AD versus Lewy body disease*(p＜0.001)


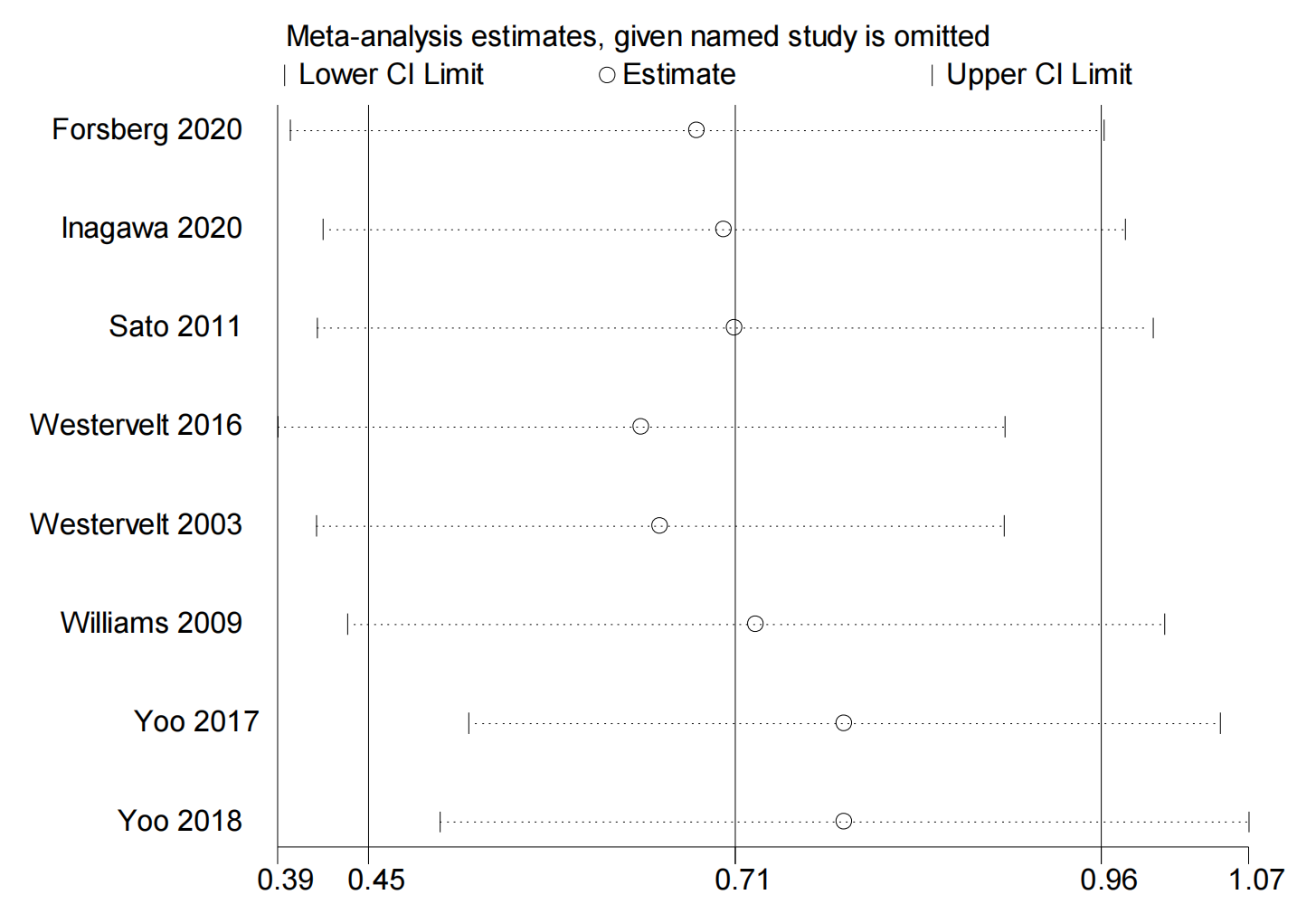


**Supplementary figure 10:** *sensitivity analysis results in AD versus LBD*


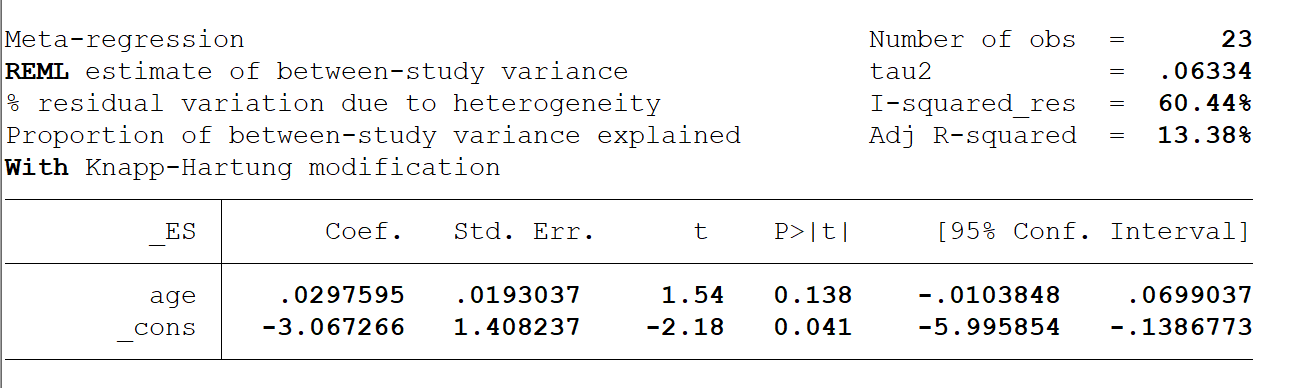


**Supplementary figure 11:** *meta-regression results in AD versus MCI on age*


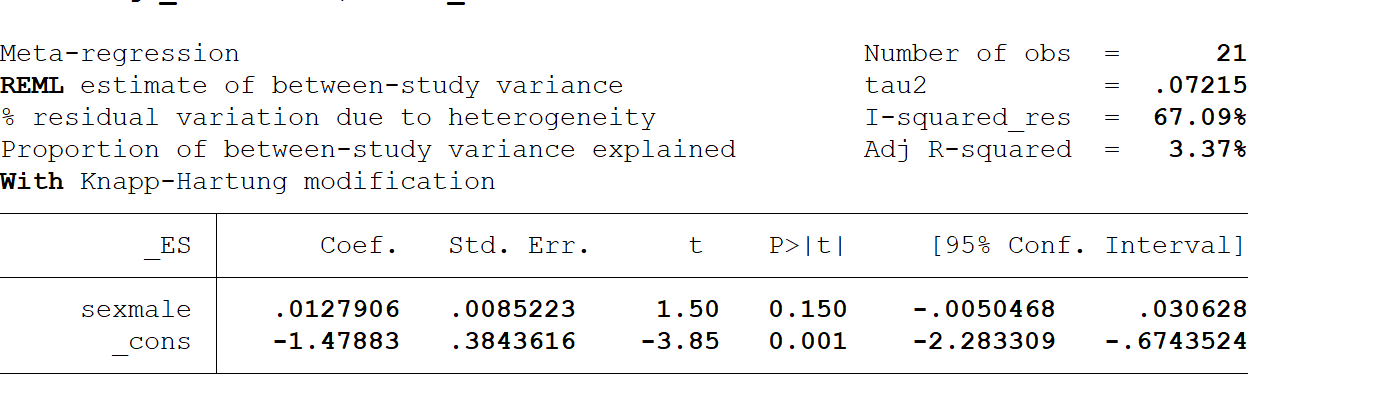


**Supplementary figure12:** *meta-regression results in AD versus MCI on sex (male%)*


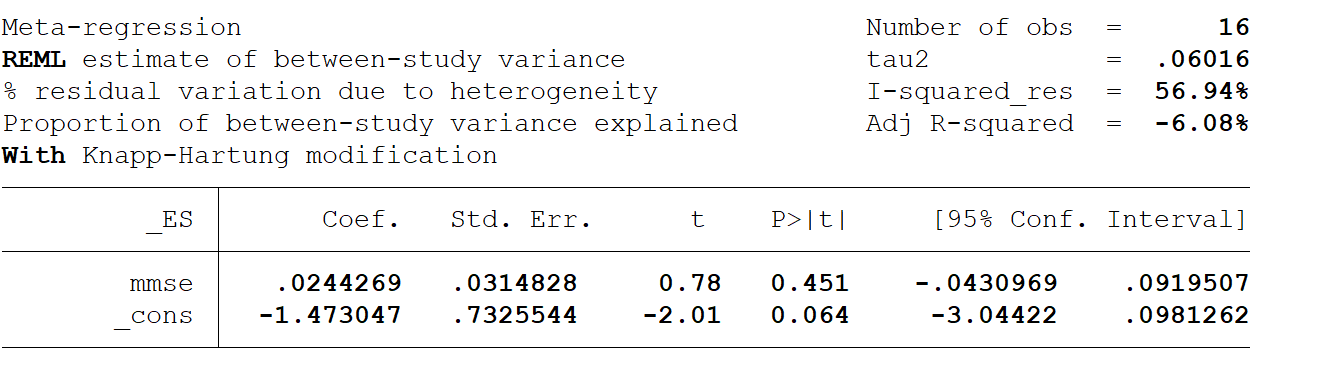


**Supplementary figure13:** *meta-regression results in AD versus MCI on MMSE scores.*


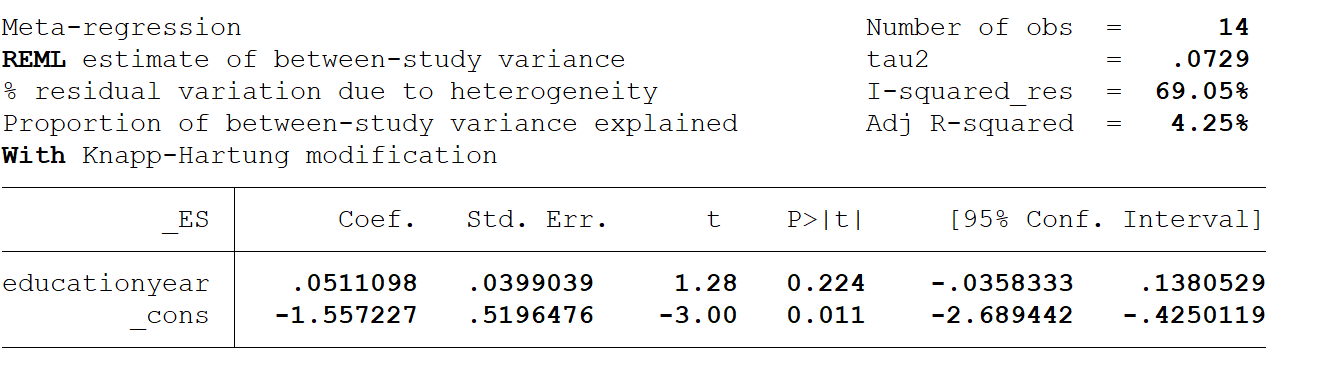


**Supplementary figure 14:** *meta-regression results in AD versus MCI on education years*


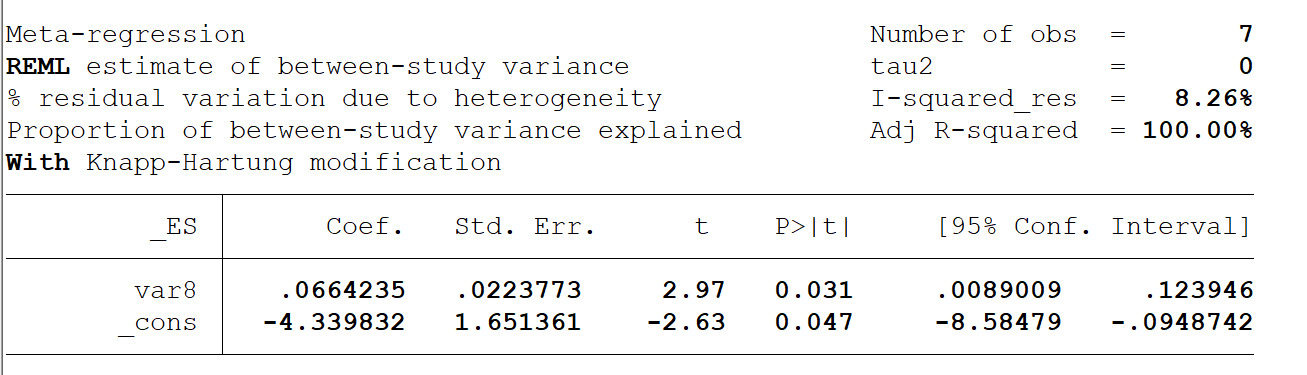


**Supplementary figure 15:** *meta-regression results in AD versus LBD on age*


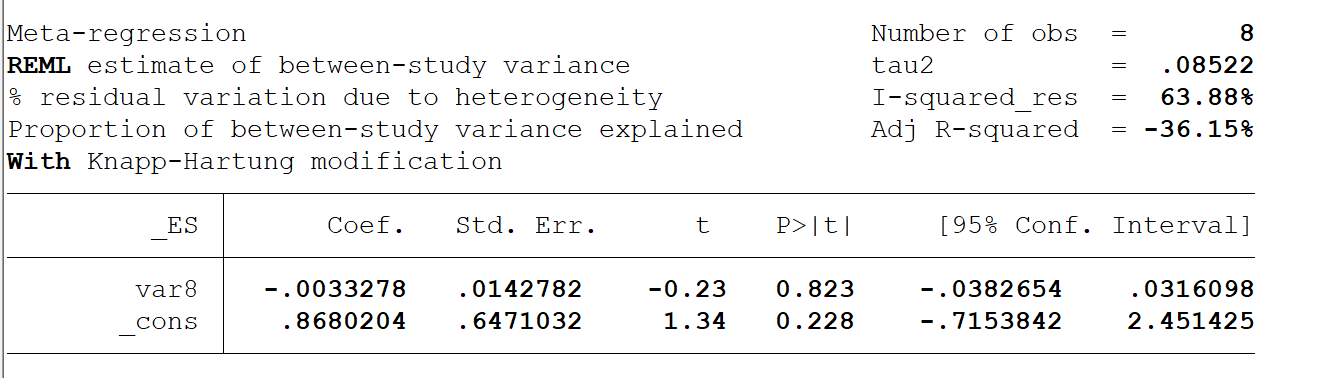


**Supplementary figure 16:** *meta-regression results in AD versus LBD on sex (male%)*


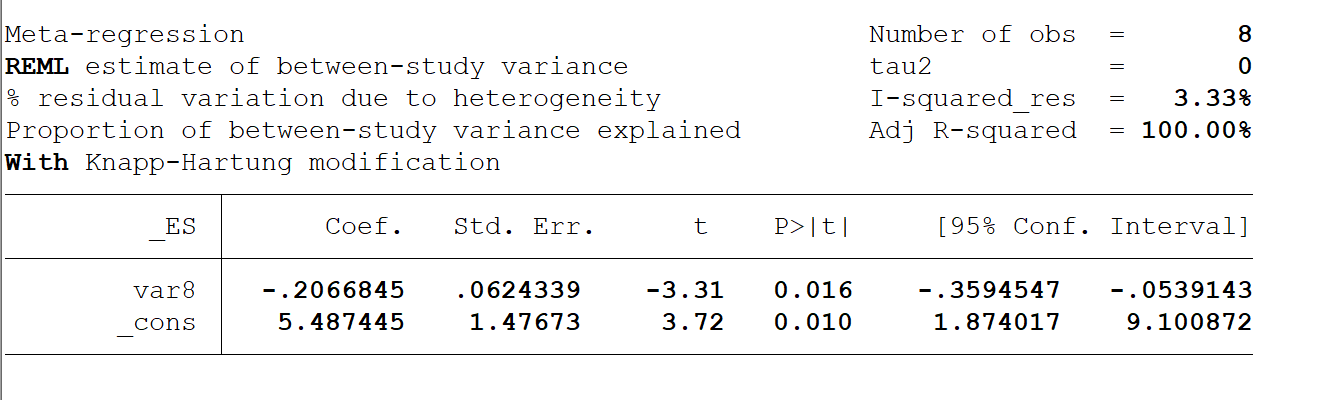


**Supplementary figure 17:** *meta-regression results in AD versus LBD on MMSE scores*


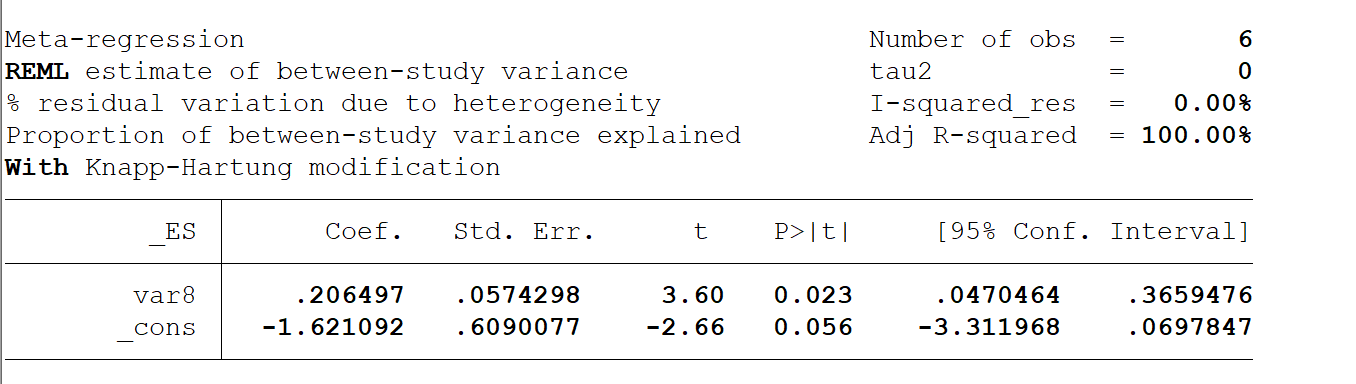


**Supplementary figure 18:** *meta-regression results in AD versus LBD on education year*
